# Facilely Synthesis of MOF-Derived Nickel Phyllosilicate Nanosheet Assembly and Its Application in Robust, Toughened and Wear-Resistant Epoxy Composites

**DOI:** 10.3390/polym18182206

**Published:** 2026-09-10

**Authors:** Dongliang Zhang, Weilong Chen, Hengzhi Zhou, Jinian Yang

**Affiliations:** 1 School of Ocean Engineering, Guangzhou Maritime University, Guangzhou 510725, China; 2 School of Materials Science and Technology, Anhui University of Science and Technology, Huainan 232001, China; 3 School of Materials Science and Engineering, Nanjing Institute of Technology, Nanjing 211167, China

**Keywords:** nickel phyllosilicate nanosheet assembly, epoxy composites, mechanical property, thermal stability, wear mechanism

## Abstract

Epoxy (EP) exhibits inherent brittleness and poor wear resistance, limiting its service under long-term frictional conditions. Two-dimensional layered nanofillers are promising candidates for tribological enhancement, yet uniform filler dispersion and robust filler–matrix bonding remain challenging. In this study, nickel phyllosilicate nanosheet assemblies (NiPS−NA) were synthesized via a two-step hydrothermal route using Ni-MOF as a sacrificial template and blended into the EP matrix to fabricate composites. A series of characterizations were carried out to evaluate the structure–property relationships of EP/NiPS–NA composites. Loosely stacked NiPS–NA nanosheets disperse uniformly within the EP matrix and comprehensively improve the comprehensive properties of EP/NiPS–NA composites. As NiPS–NA loading increases, elongation at break rises to a peak value of 6.0% at 3% filler content, representing a 43.3% improvement over neat EP, whereas tensile strength increases steadily with only minor variations in elastic modulus. Incorporated NiPS–NA suppresses thermal weight loss, elevates the high-temperature char yield and raises the activation energy for thermal decomposition. Notably, the composite containing 5% NiPS–NA delivers the lowest wear rate of 1.02 × 10^−5^ mm^3^/N·m, an 89.4% reduction relative to unfilled EP. Extensive examinations of worn surfaces further clarify the NiPS–NA-mediated wear mechanism. This work offers a facile three-in-one strategy for preparing high-performance wear-resistant polymeric composites.

## 1. Introduction

Polymers are widely used as structural and functional materials in many industrial sectors, including aerospace, automotive, electronics, construction and consumer goods. Their popularity stems from a set of inherent merits compared with metallic and inorganic materials, including light weight, self-lubricity, outstanding chemical and corrosion resistance, easy processing and low production cost [1]. Nevertheless, weak intermolecular forces render polymer molecular chains susceptible to sliding and fracture under friction-scratching conditions, triggering surface peeling and surface damage, which severely restricts their practical deployment under persistent abrasive service conditions [2]. Among various polymer candidates, epoxy resin (EP) stands out as a promising high-performance thermosetting matrix for wear-resistant composites, benefiting from its high mechanical strength, excellent dimensional stability, reliable chemical resistance and convenient curing manufacturability [3]. However, highly cross-linked EP networks bring intrinsic brittleness, making neat EP vulnerable to fatigue-driven material loss during dry sliding, hence requiring rational filler modification to achieve satisfactory tribological performance [4]. To address this limitation, considerable efforts have been devoted to developing wear-resistant polymer-modification strategies. Compared with complex molecular-level structural regulation, incorporating inorganic nanofillers represents a cost-effective and scalable route [5]. Inorganic fillers can serve as physical barriers to suppress molecular-chain slippage, improve surface hardness and load-bearing capacity, and mitigate abrasive material removal without tedious synthetic workflows [6].

Over the past decades, a broad spectrum of two-dimensional layered nanomaterials, such as montmorillonite [7], MXenes [8], molybdenum disulfide (MoS_2_) [9], and boron nitride (BN) [10], have been adopted to enhance the tribological performance of EP composites. Layered nanomaterials are particularly attractive owing to their lamellar geometry, high specific surface area and unique interlayer-sliding characteristics, which facilitate transfer-film generation and friction reduction [11,12]. Even so, practical challenges persist: poor filler–matrix interfacial compatibility frequently induces nanofiller agglomeration, stress concentration and premature composite failure [12]. Conventional post-treatment routes including silane grafting and etching can ameliorate dispersion, yet these protocols often consume large volumes of organic solvents and involve time-consuming operations [13,14,15]; furthermore, restacking of exfoliated nanosheets commonly recurs during resin compounding and curing [14]. These bottlenecks motivate the exploration of alternative filler-fabrication strategies.

MOFs possess tunable chemical composition, high specific surface area and intrinsic ordered porous architectures, rendering them uniquely suitable as self-sacrificial templates rather than being directly applied as composite fillers [16,17]. Their periodically arranged metal nodes act as built-in nucleation sites for oriented in situ nanosheet growth; meanwhile, the inherent micro-/mesoporous framework delivers spatial-confinement effects during hydrothermal transformation [18]. Such confinement suppresses excessive crystal growth and severe nanosheet restacking, favoring loosely stacked nanosheet-assembly structures with abundant exposed active surface sites. These derived nanostructures can form sufficient physical entanglement with EP molecular chains without complicated post-modification. It is worth emphasizing that pristine MOF crystals are not ideal direct fillers for EP composites, despite their attractive structural features. Bare MOF materials generally exhibit unsatisfactory thermal stability under frictional heating, and their intrinsic brittleness, together with poor chemical compatibility with EP matrices, readily trigger particle agglomeration and defective interfacial regions, which become stress-concentration points and degrade composite mechanical and anti-wear performance. Therefore, converting MOF templates into thermally stable MOF-derived inorganic derivatives becomes a rational technical route.

Our previous work has verified that the assembly morphology of nickel phyllosilicate exerts decisive influences on composite performances; flower-shaped nickel phyllosilicate delivers better comprehensive reinforcement compared with lamellar counterparts [19]. Inspired by these findings, this work adopted a two-step hydrothermal reaction to prepare NiPS–NA nanosheet assemblies via Ni-MOF sacrificial-template conversion, which were homogeneously blended into an EP matrix to fabricate EP/NiPS–NA composites. The phase structure, micromorphology and chemical composition of NiPS–NA were systematically characterized. Subsequently, we comprehensively investigated how NiPS–NA loading modulates the structure–property relationships of as-prepared composites under starved-lubrication dry-sliding conditions, and clarified the corresponding wear mechanism. This research provides a MOF-templated strategy for regulating layered-nanosheet assembly and offers a feasible pathway toward high-performance tribological polymer composites.

## 2. Experimental

**Scheme 1:**
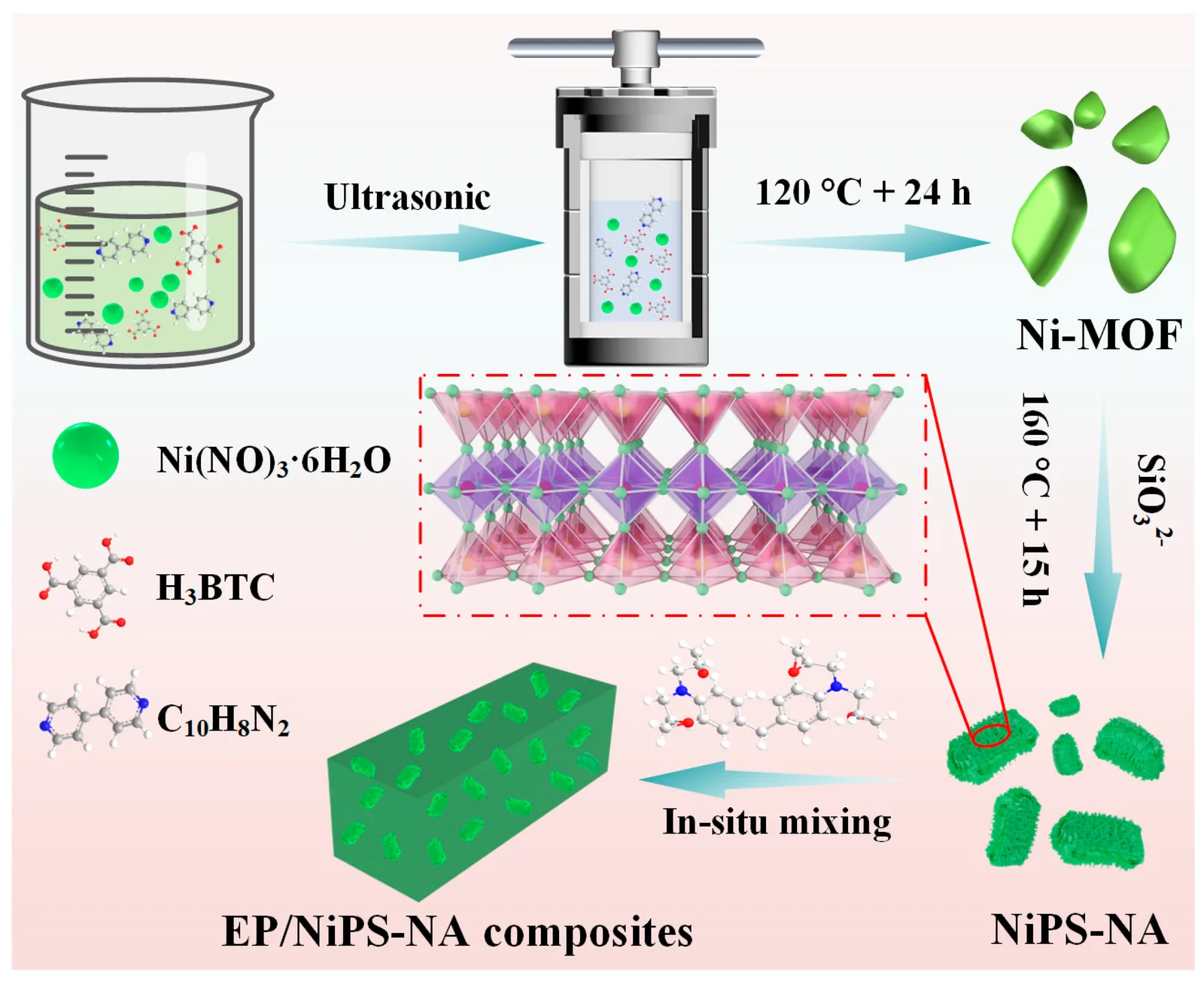
Schematic of the experimental workflow for synthesizing NiPS–NA filler and fabricating EP/NiPS–NA composites.

Detailed information on the used chemicals, raw materials, characterization techniques, and the design and fabrication of EP/NiPS–NA composites are provided in the Supplementary Materials, and the sample preparation process is schematically illustrated in Scheme 1, while the sample formulae are shown in Table S1.

## 3. Results and Discussion

### 3.1. Characterizations of NiPS–NA

**Figure 1:**
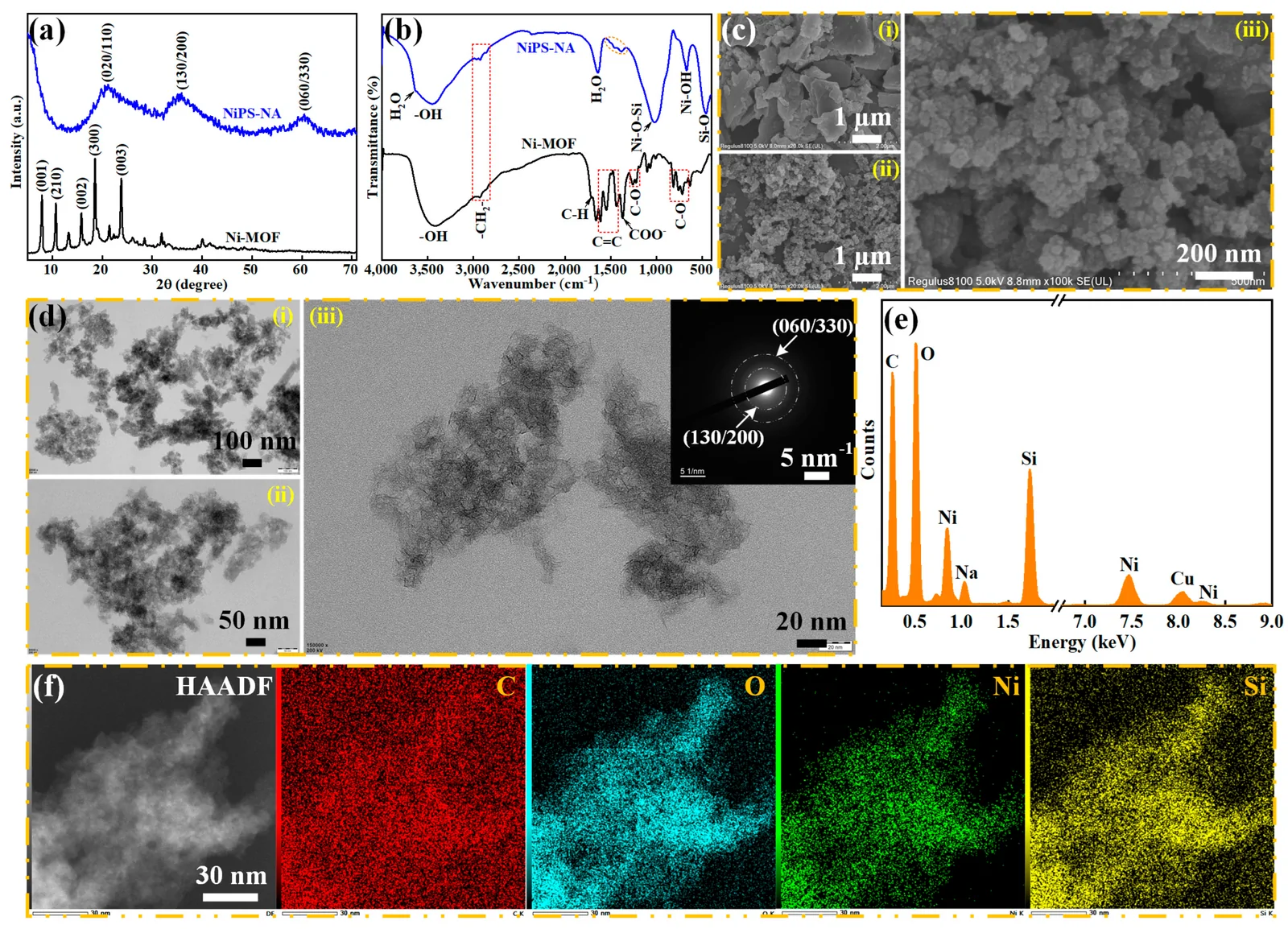
(**a**) XRD and (**b**) FT-IR results of Ni-MOF and NiPS–NA; (**c**) SEM images of Ni-MOF (i), NiPS–NA with low (ii) and high magnifications (iii); (**d**) TEM images of NiPS–NA with inserted SAED pattern, (**e**) EDS pattern and (**f**) HAADF image and elemental mappings of NiPS–NA.

As illustrated in Scheme 1, Ni-MOF is first synthesized via a facile hydrothermal reaction involving nickel (Ni) ions and the organic ligands H_3_BTC and 4,4′-bipyridine. The 2D planar layers are composed of divalent Ni ions and partially deprotonated H_3_BTC, while 4,4′-bipyridine acts as a bridging ligand to connect these layers, thereby forming a 3D porous skeleton [20]. Subsequently, these as-synthesized plate-like Ni-MOF precursors with irregular shapes are employed as Ni sources to react with SiO_3_^2−^ anions under hydrothermal conditions a second time. During this process, organic ligands are gradually substituted by inorganic [SiO_4_]^2−^ units through chemical etching, concomitantly leading to the in situ formation of abundant NiPS–NAnosheets that adhere to the MOF frameworks. The removal of organic ligands, specifically 4,4′-bipyridine, prevents the as-synthesized NiPS from retaining its original plate-like morphology. Instead, the material evolves into a loosely packed nanosheet structure that inherits the porous architecture of the Ni-MOF template, i.e., NiPS–NA. Systematic characterizations were conducted to verify the above inference, and the results are presented in Figure 1.

Figure 1a shows the XRD patterns of Ni-MOF and NiPS–NA. Sharp characteristic diffraction peaks can be seen in the XRD curve of Ni-MOF. Specifically, the five diffraction signals located at 2θ = 7.9°, 10.7°, 15.82°, 18.54° and 23.78° correspond to the (001), (201), (002), (300) and (003) crystal planes, respectively. Several weak secondary peaks are also detected, matching data from prior literature [21]. In contrast, the diffraction profile of NiPS–NA is vastly different from that of Ni-MOF. All diffraction peaks belonging to Ni-MOF vanish, and three obvious (00*L*) and (*hk*0) characteristic peaks appear, consistent with typical features of two-dimensional layered materials [22]. The diffraction band near 2θ = 19.8° is attributed to the (020/110) plane, verifying the formation of phyllosilicate rather than turbostratic nickel hydroxide [22]. The peak at approximately 2θ = 60° corresponds to the (060/330) plane, confirming a tri-octahedral smectite structure [23], and the d-spacing of the asymmetric peak around 2θ = 34° fits the (130/200) reflection of smectite [24]. Overall, these results prove the generation of willemseite, Ni_3_Si_4_O_10_(OH)_2_, a Ni-montmorillonite analog, whose layered structure consists of one Ni-O octahedral sheet sandwiched between two Si-O tetrahedral sheets [22]. A schematic molecular structure is displayed in Scheme 1. These XRD results confirm complete hydrothermal transformation from Ni-MOF to NiPS–NA. The broadened XRD peaks reveal poor long-range stacking order in the aggregates, which arises from MOF-template spatial confinement that produces randomly oriented, loosely stacked nanosheets and eliminates low-angle basal-stacking diffraction signals. However, local crystallinity persists within individual nanosheet domains, as supported by the distinguishable SAED rings matching the lattice planes of willemseite. The co-existence of broad XRD reflections and discrete SAED rings is characteristic of poorly crystalline layered assemblies, i.e., long-range stacking periodicity disappears over the micron-scale aggregate, yet short-range atomic-scale lattice ordering remains in each nanosheet fragment, instead of yielding highly ordered bulk NiPS single crystals.

The FT-IR spectra of Ni-MOF and NiPS–NA are displayed in Figure 1b, revealing structural differences between the two samples. The Ni-MOF spectrum exhibits an intense band at 3413 cm^−1^, assigned to the stretching vibration of adsorbed -OH groups from water. Twin peaks at 2931 cm^−1^ and 2861 cm^−1^ correspond to symmetric and asymmetric stretching of methylene (-CH_2_) groups, respectively, whereas the band at 1712 cm^−1^ belongs to C-H stretching vibrations. Intense absorption bands at 1612, 1550 and 1440 cm^−1^ originate from benzene ring C=C skeletal vibrations of the Ni-MOF organic ligand [20]. Moreover, the peak at 763 cm^−1^ (C-H out-of-plane bending) and the overtone band at 1716 cm^−1^ further confirm benzene ring structures. Signals at 819 and 717 cm^−1^ are characteristic of 1,3,5-trisubstituted benzene rings, and a strong peak at 1367 cm^−1^ corresponds to carboxylate (COO^−^) groups [20]. Additional C-O stretching bands at 1222 and 1265 cm^−1^ prove the existence of carboxylic acid moieties within the Ni-MOF framework [20]. In contrast, the FT-IR spectrum of NiPS–NA shows dramatic structural changes. The broad, dominant band centered at 3415 cm^−1^ together with a shoulder peak at 3473 cm^−1^ is attributed to stretching vibrations of crystalline water. A new band at 1635 cm^−1^ arises from the bending vibration of physically adsorbed water [25]. Characteristic peaks at 1018 cm^−1^ and 457 cm^−1^ correspond to the stretching vibrations of Ni-O-Si [26] and Si-O bonds [27], respectively. A single absorption peak at 673 cm^−1^ is assigned to lattice Ni-OH stretching within the phyllosilicate skeleton [28], which further confirms successful NiPS–NA synthesis.

The morphologies of Ni-MOF and as-derived NiPS–NA were characterized, as displayed in Figure 1c. As shown in Figure 1c(i), the Ni-MOF precursor possesses a typical aggregated two-dimensional structure stacked by plenty of irregular micron-scale nanosheets with smooth surfaces and distinct edges. These petal-like folded aggregates are the typical morphology of self-assembled MOFs. Their unique 2D layered structure supplies abundant active sites and confined spaces for subsequent hydrothermal conversion. After the hydrothermal treatment, the original sheet-shaped Ni-MOF structure fully transforms. Low-magnification SEM micrographs [Figure 1c(ii,iii)] demonstrate the complete disappearance of the Ni-MOF layered skeleton; the resultant NiPS–NA forms compact micron clusters assembled from tiny nanosheets that interlink to build a continuous porous network. TEM images [Figure 1d(i,ii)] further verify such randomly stacked nanosheet morphology, consistent with SEM results. The high-resolution TEM mass-thickness contrast image [Figure 1d(iii)] confirms that crystalline lattices are formed via stacking and assembly of phyllosilicate nanosheets, proving the thorough transformation of Ni-MOF sheets into polycrystalline nanosheet aggregates. In the SAED pattern, clear diffraction rings with lattice spacings of 0.261 nm and 0.153 nm match the (13/20) and (06/33) crystal planes of NiPS, revealing the polycrystalline nature of NiPS–NA, which agrees well with the broadened XRD peaks. The EDS spectrum in Figure 1e presents strong characteristic signals of C (0.26 keV) and O (0.51 keV). Three obvious nickel peaks appear at 0.85, 7.48 and 8.74 keV, while silicon gives a signal at 1.74 keV. A weak Na peak at 1.02 keV originates from residual sodium hydroxide, and a minor Cu signal at 8.04 keV comes from the TEM supporting grid. Elemental mapping in Figure 1f shows a homogeneous distribution of C, O, Ni and Si across the whole sample without elemental agglomeration or segregation. Such uniform element distribution proves sufficient reaction between Ni ions released from Ni-MOF and silicate ions under hydrothermal conditions. Carbon residues decomposed from organic ligands are evenly embedded within the inorganic framework, yielding compositionally homogeneous NiPS–NA.

**Figure 2:**
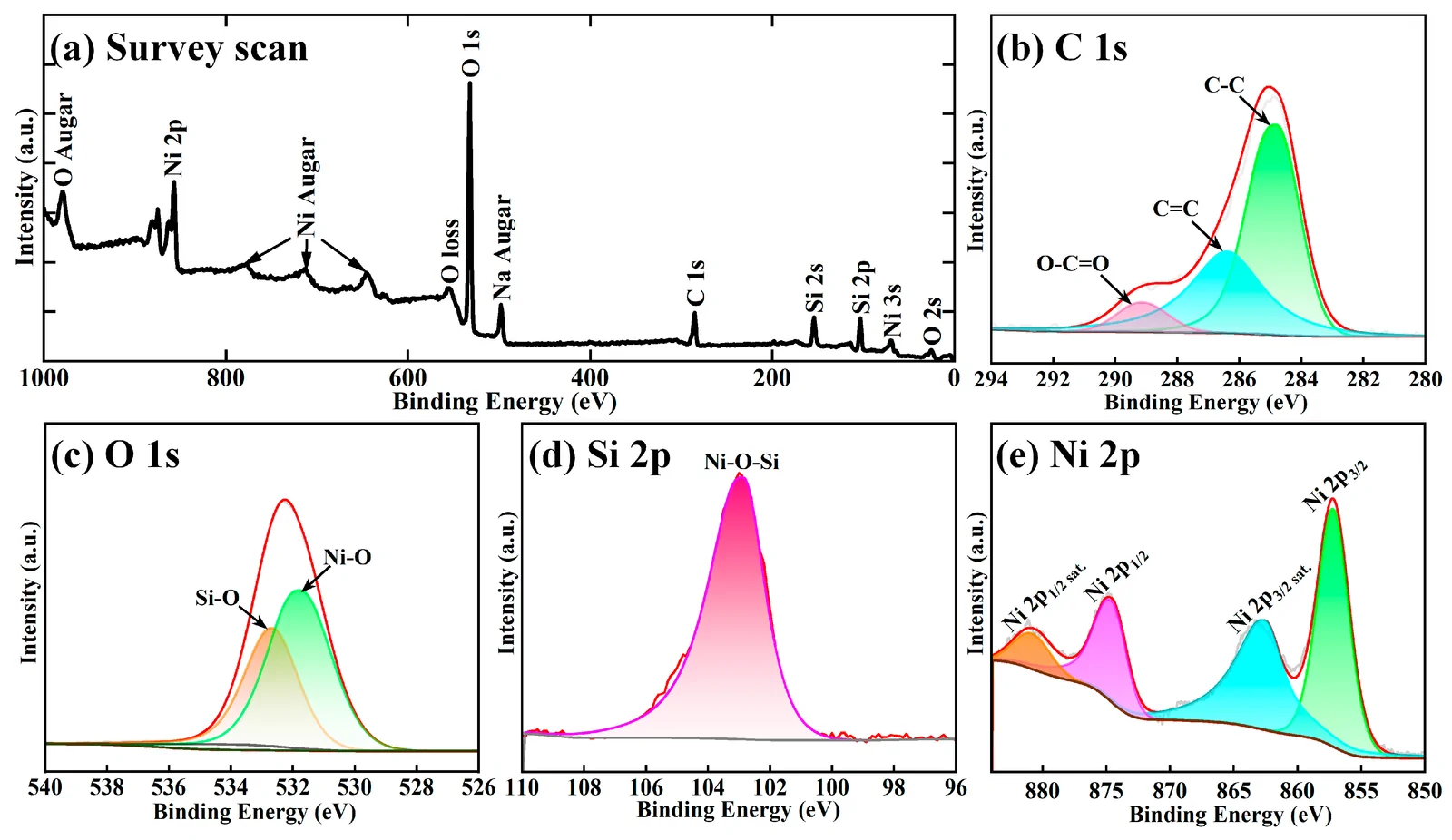
XPS measurements of NiPS–NA: (**a**) full survey spectrum, (**b**–**e**) high-resolution spectra of elemental components.

To further elucidate the elemental composition, chemical coordination environments and interfacial bonding states of as-prepared NiPS–NA, XPS characterization was carried out, and the corresponding results are shown in Figure 2. The full survey spectrum (Figure 2a) verifies the coexistence of Ni, Si, O and C in NiPS–NA; their atomic percentages are 8.54 at% (Ni 2p), 17.90 at% (Si 2p), 52.98 at% (O 1s) and 19.17 at%, respectively (C 1s). The carbon signals mainly stem from carbon residues generated by thermal decomposition of Ni-MOF organic ligands during hydrothermal transformation. High-resolution XPS spectra were collected to analyze elemental valence states and bonding configurations. As displayed in Figure 2b, the C 1s spectrum can be deconvoluted into three peaks centered at 284.9, 286.4 and 289.1 eV, assigned to C–C/C=C, C–O and O–C=O bonds separately. These signals are typical carbon residues from MOF ligand decomposition, in line with prior research on MOF-derived inorganic hybrids [29]. The O 1s spectrum (Figure 2c) was fitted into two dominant peaks at 531.8 eV and 532.7 eV, corresponding to Ni–O bonds within octahedral Ni(OH)_6_ layers and Si–O bonds in tetrahedral SiO_4_ units of phyllosilicate [30]. For the Si 2p spectrum (Figure 2d), a sharp peak at 102.9 eV is unambiguously attributed to Ni–O–Si linkages [31]. The Ni 2p spectrum (Figure 2e) contains two groups of spin–orbit peaks plus two satellite signals. The primary peaks at 857.2 eV and 874.8 eV correspond to Ni 2p_3/2_ and Ni 2p_1/2_, while satellite bands at 862.7 eV and 881.0 eV are characteristic shake-up peaks of divalent Ni(II) [30]. The existence of these bonds reveals strong chemical coupling between Ni-centered octahedral sheets and Si-based tetrahedral sheets, the core structural feature of nickel phyllosilicate. In addition, no signals of metallic Ni^0^ or Ni^3+^ are detected, confirming that nickel in NiPS–NA only exists in the +2-oxidation state, consistent with the standard coordination configuration of nickel phyllosilicate. Taken together, XPS data combined with the above characterizations offer solid, full proof that pure-phase NiPS–NA is successfully fabricated via the MOF-templated hydrothermal method.

### 3.2. Morphological Structures of EP/NiPS–NA Composites

**Figure 3:**
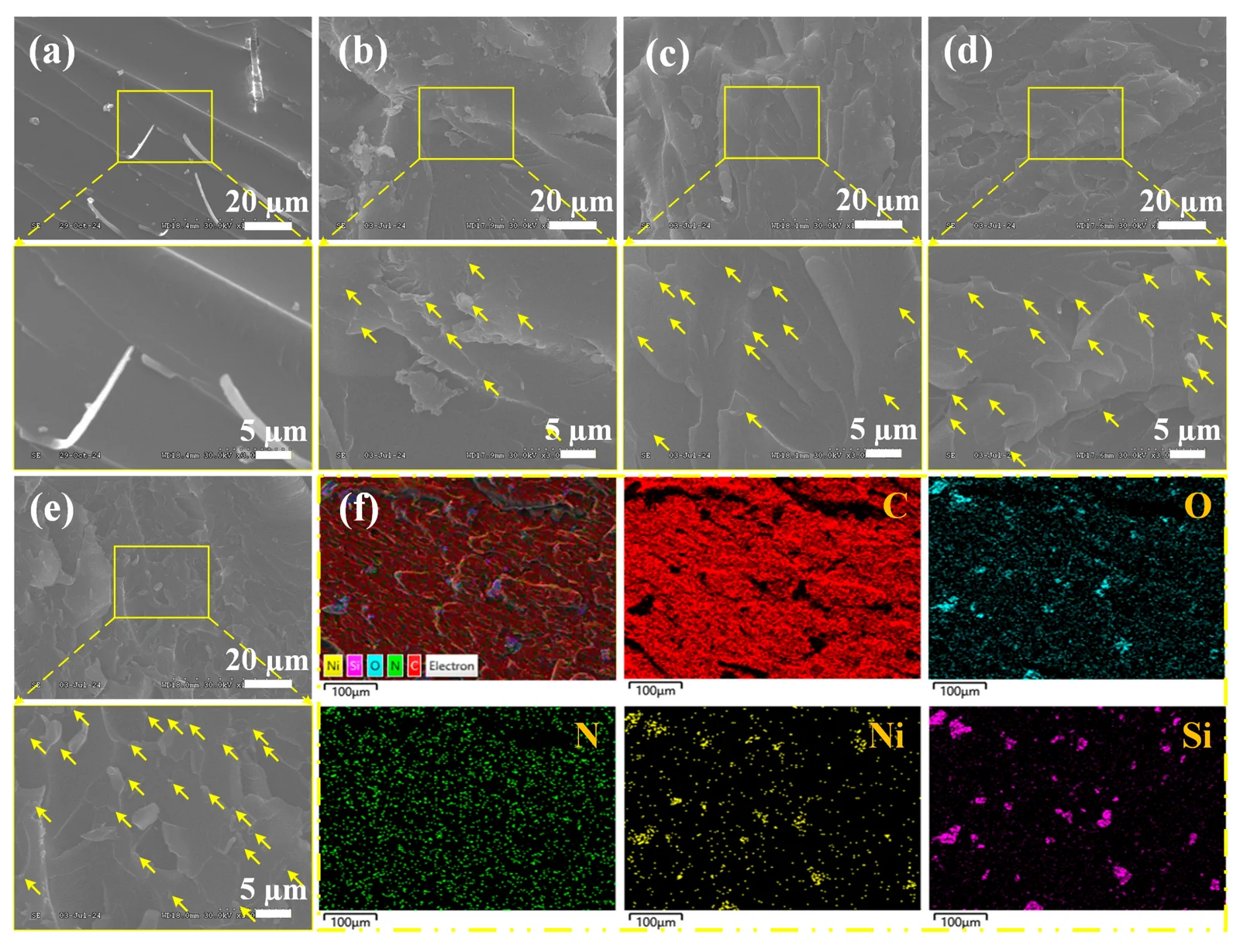
SEM micrographs of fractured morphologies for EP/NiPS–NA composites with different loadings: (**a**) 0%, (**b**) 1%, (**c**) 3%, (**d**) 5% and (**e**) 7%; (**f**) element mappings of the composites containing 7% NiPS–NA.

The dispersion state, interfacial compatibility and interfacial adhesion of NiPS–NA within the EP matrix were systematically evaluated through SEM observation of tensile-fractured surfaces. Figure 3 displays the micro-morphologies of neat EP and its composites at magnifications of 1000× and 3000×, respectively. As presented in Figure 3a, the fracture surface of neat EP is notably smooth, flat and featureless, accompanied by typical river-like patterns and straight continuous cleavage steps. This morphology is characteristic of brittle fracture behavior, which arises from the highly crosslinked thermosetting EP network that restricts macromolecular chain mobility and provides limited resistance to crack propagation [32]. Once microcracks initiate under external tensile stress, they propagate rapidly and unimpeded throughout the matrix, resulting in catastrophic brittle failure with minimal energy dissipation. In sharp contrast, the incorporation of NiPS–NA nanofillers dramatically remodels the fracture morphology of the EP matrix. As illustrated in Figure 3b, the fracture surface becomes considerably rougher, with numerous protuberances, wrinkles, folds and distorted crack paths distributed uniformly across the section. With increasing NiPS–NA content (Figure 3c–e), surface roughness and morphological complexity are further enhanced. This distinct evolution indicates that well-dispersed NiPS–NA act as effective physical barriers to interrupt, deflect and branch propagating cracks [33], thereby transforming the fracture mode from typical brittle fracture to a ductile-like state. It is well established that both internal defects and secondary filler phases dominate crack growth. For neat EP, crack initiation occurs at internal defect sites that concentrate stress, while EP/NiPS–NA composites are affected by both defects and dispersed NiPS–NA particles. These fillers provide a large specific surface area to form abundant polymer–filler interfacial bonds [34]. Additional cracks with extended propagation paths form before fracture, consuming more strain energy and reducing matrix brittleness.

High-magnification SEM images further confirm the favorable dispersion and strong interfacial interaction between NiPS–NA and EP. NiPS–NA occurs as tiny nanosheet assemblies with moderate cluster sizes, and no obvious interfacial debonding, voids, or filler pull-out can be detected at the filler–matrix interfaces. Under the adopted mixing and curing conditions, NiPS–NA assemblies are uniformly dispersed within the EP matrix despite random sizes and morphologies, and this dispersion state is regarded as satisfactory. Notably, severe agglomeration is absent even at 5% NiPS–NA loading, which originates from the distinctive hierarchical nanosheet-assembled architecture of MOF-derived NiPS–NA. Its large specific surface area and abundant surface hydroxyl groups facilitate sufficient physical entanglement and interfacial interactions with epoxy chains, mitigating the intrinsic polarity mismatch between inorganic nanofillers and organic polymers [35]. These observations verify favorable wettability and interfacial compatibility between hierarchical NiPS–NA and EP molecular chains even at relatively high filler contents. The corresponding EDS elemental mappings (Figure 3f) intuitively show homogeneous distribution of C, O, N, Ni and Si over the fracture surface, without evident element aggregation or segregation. Such uniform dispersion is essential for constructing abundant filler–matrix interfaces, which enable efficient stress transfer and suppress rapid crack propagation upon mechanical loading [36]. Undoubtedly, this desirable dispersion and robust interfacial adhesion provide a solid microstructural basis for the simultaneous enhancement of mechanical strength, toughness and wear resistance of EP/NiPS–NA composites.

### 3.3. Mechanical Properties of EP/NiPS–NA Composites

**Figure 4:**
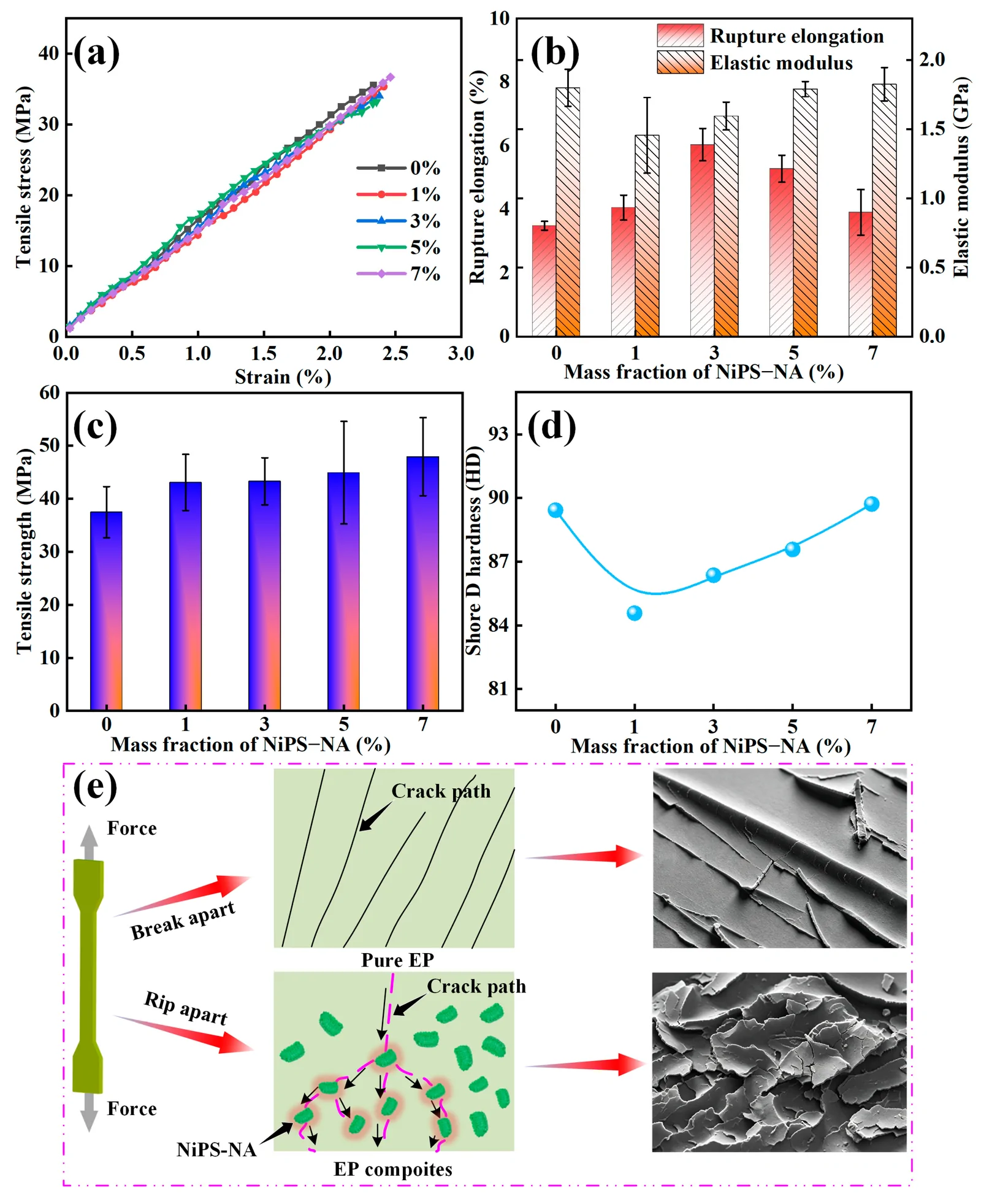
The typical stress–strain plots obtained under quasi-state tensile testing (**a**), the variations in rupture elongation and elastic modulus (**b**), tensile strength (**c**) and Shore D hardness (**d**) with increased NiPS–NA content; (**e**) possible strengthening and toughening mechanism of EP/NiPS–NA composites.

The mechanical properties of neat EP resin and its corresponding composites were quantitatively characterized via quasi-static tensile tests. Figure 4 shows the typical tensile stress–strain curves, as well as the variations in tensile strength, elastic modulus, and elongation at break as functions of NiPS–NA loading. As clearly observed in Figure 4a, all tested specimens exhibit similar tensile behavior, and their stress–strain curves can be divided into two stages before fracture. The tensile stress increases rapidly in an approximately linear manner at small strains, followed by slight non-linear deformation without a distinct yield plateau, and finally fractures abruptly without obvious warning signs. All tensile responses confirm that both neat EP and its composites retain the typical brittle fracture characteristics under uniaxial tension. In other words, the incorporation of NiPS–NA does not fundamentally change the intrinsic fracture mode of the epoxy matrix; however, it significantly modulates the key mechanical parameters in a filler loading-dependent manner. As illustrated in Figure 4b, the elongation at break of the composites first increases and then decreases with increasing NiPS–NA content, while the elastic modulus shows an approximately opposite trend. Specifically, the elongation at break reaches a maximum value of 6.0% at 3% NiPS–NA, which is 43.3% higher than that of the control sample (3.4%). This result is noteworthy and differs from our previous observations, in which phyllosilicate fillers normally reduced the ductility of EP, leading to a continuous decrease in elongation at break with increasing filler content [37]. It is well established that both elongation at break and elastic modulus are closely governed by the mobility of polymer chains under external loading. Higher chain mobility favors larger elongation at break, while restricted mobility gives rise to a higher elastic modulus. On the one hand, NiPS–NA can insert into the interchain regions of the EP matrix, weakening excessive intermolecular interactions and promoting the mobility of molecular segments. On the other hand, the large surface area of NiPS–NA induces physical entanglement with EP chains, which constrains chain mobility. These two competing effects dominate the overall mechanical response, depending on the filler loading. At a NiPS–NA content lower than 3%, the chain-mobility-enhancement effect prevails [38], leading to improved elongation at break and a slightly reduced elastic modulus. With further increasing NiPS–NA addition, the entanglement-induced chain-restriction effect becomes dominant, accompanied by the transition from chain-mobility promotion to rigidity enhancement, resulting in a gradual decrease in elongation at break and a recovery of the elastic modulus [16]. As shown in Figure 4c, the tensile strength increases monotonically with increasing NiPS–NA loading. The tensile strength reaches a maximum of 47.94 MPa at 7% NiPS–NA, representing an improvement of 21.8% compared with neat EP (37.47 MPa). This result clearly demonstrates the remarkable reinforcing effect of NiPS–NA on EP. The enhanced tensile strength is mainly attributed to the uniform dispersion of fine NiPS–NA nanosheet assemblies within the resin matrix. The robust interfacial adhesion and effective physical entanglement between NiPS–NA and resin molecular chains promote the formation of a rigid filler network in the polymer matrix. Such a network enables efficient stress transfer across the filler–matrix interface, enabling the composite to bear higher external loads and thus contributing to the significantly improved tensile strength of EP/NiPS–NA composites [39]. As presented in Figure 4d, the Shore D hardness of the composites shows a similar trend to the elastic modulus with increasing NiPS–NA content. The addition of 1% NiPS–NA exerts little positive effect on hardness, which can be explained by the enhanced ductility of the EP matrix at low filler loading, as discussed above. In contrast, further increasing the filler content introduces more rigid inorganic components, which gradually reinforce the bulk material and enhance the overall rigidity. This mechanism reasonably explains the continuous increase in hardness observed in the EP composites. Based on the above morphological and mechanical analyses, a plausible toughening and strengthening mechanism for EP/NiPS–NA composites is proposed and illustrated in Figure 4e.

### 3.4. Thermal Stability of EP/NiPS–NA Composites

**Figure 5:**
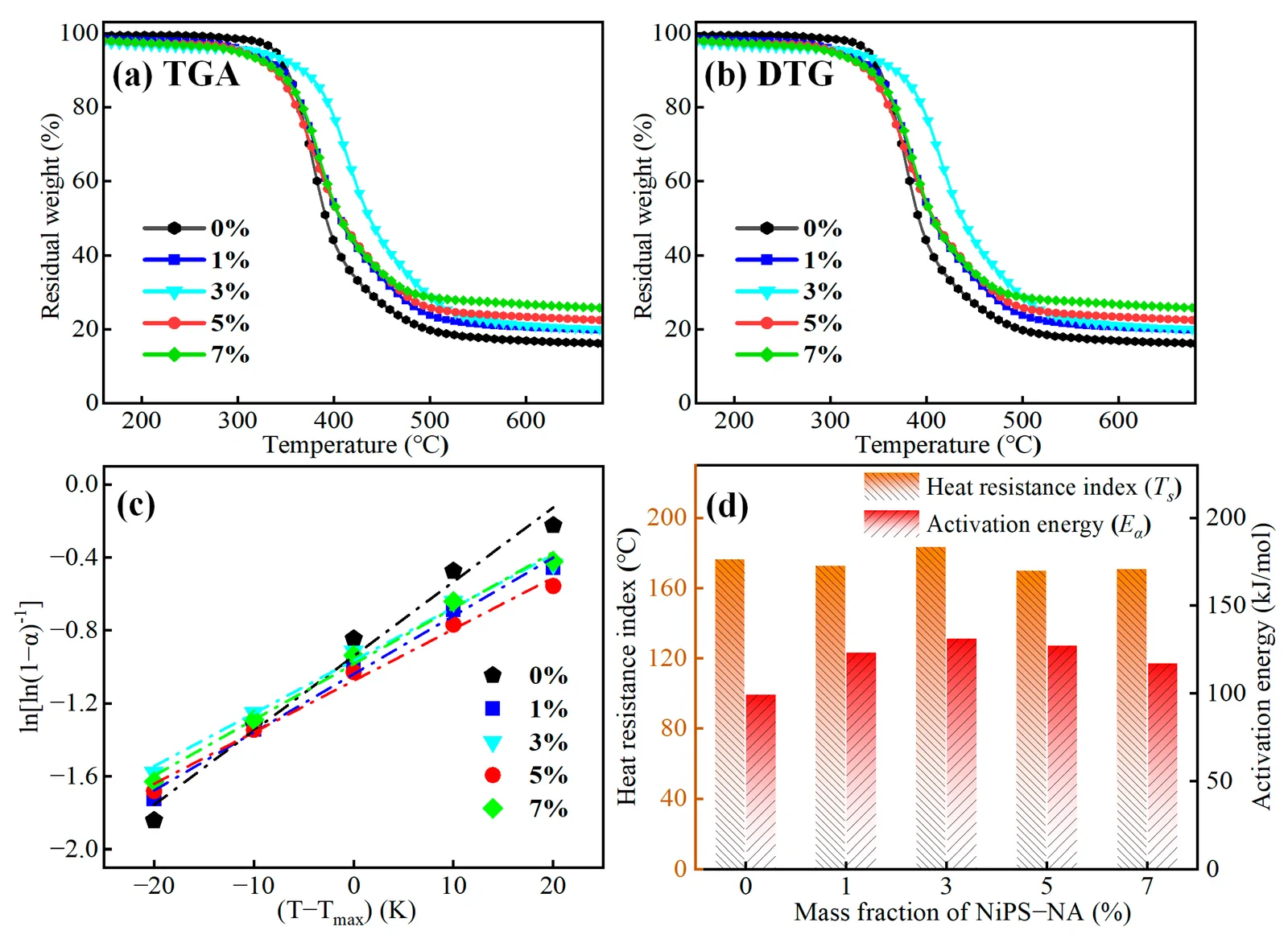
Thermal properties of EP/NiPS–NA composites: (**a**) residual weight and (**b**) weight loss rate as a function of increasing temperature, (**c**) fitting plots based on Horowitz and Metzger equation and (**d**) heat resistance indices and activation energies versus NiPS–NA content.

The thermal stabilities of neat EP and composites with different mass fractions of NiPS–NA were systematically investigated via thermogravimetric analysis under a nitrogen atmosphere. The corresponding thermogravimetric (TGA) curves, derivative thermogravimetric (DTG) thermograms, and relevant key thermal parameters are presented in Figure 5 and Table S2. As shown in Figure 5a, neat EP exhibits a typical single-step thermal decomposition profile, with the majority of weight loss occurring within a narrow temperature window. This behavior is characteristic of a first-order solid-state reaction [40], which is dominated by random chain scission and pyrolysis of the highly cross-linked EP network during thermal decomposition [41]. The DTG curve (Figure 5b) further confirms this decomposition mode, displaying a single sharp and intense decomposition peak. Consistent with previous reports by Wan et al. [42] and our prior studies [16,43,44], this one-step decomposition is universally observed for fully cured EP systems. After incorporating NiPS–NA nanofillers at various loadings, all composites retain nearly identical one-step decomposition trends, indicating that the addition of NiPS–NA does not fundamentally alter the intrinsic thermal-decomposition mechanism of the resin matrix. Nevertheless, critical thermal characteristic parameters show clear filler-loading-dependent evolution, as presented in Figure 5d and summarized in Table S2. Under programmed heating conditions, neat resin possesses an initial decomposition temperature (*T_5%_*) of 322.7 °C. With increasing NiPS–NA content, the *T_5%_* values of the composites decrease to the range of 299.3–321.5 °C. Such a moderate reduction is attributed to the catalytic effect of Ni^2+^ species in the NiPS–NA structure, which lowers the energy barrier for the early-stage cleavage of C–O and C–C bonds in the EP cross-linked network [45]. Meanwhile, the *T_s_* values of the composites remain within 169.9~178.6 °C, showing negligible deviation from those of neat EP. This result demonstrates that the introduction of NiPS–NA exerts no significant adverse effect on the long-term intrinsic heat resistance of the material, ensuring its service safety under conventional operating-temperature conditions. Furthermore, neat EP has a maximum decomposition temperature (*T_max_*) of 379.6 °C and a corresponding maximum mass-loss rate (*dW*/*dT*)_max_ of 1.12 K/%. Depending on filler content, the composites exhibit moderately higher *T_max_* (373.6~410.7 °C) and considerably lower (*dW*/*dT*)_max_ values, demonstrating that incorporated NiPS–NA improves the thermal stabilization of EP during the main decomposition stage. This improvement arises from the following two synergistic mechanisms: (1) uniformly dispersed NiPS–NA fillers form strong interfacial interactions with resin chains via hydrogen bonding and physical entanglement, effectively restricting the thermal motion and segmental mobility of polymer chains during decomposition [46]; and (2) abundant NiPS–NA particles construct a dense physical-barrier network within the matrix, creating tortuous diffusion pathways that significantly hinder the escape of pyrolysis products and slow down the overall mass-transfer rate [47].

The thermal decomposition activation energy (*E_α_*) of each sample was calculated using the Horowitz–Metzger method according to the fitting results shown in Figure 5c and Table S3, and the corresponding values are summarized in Table S2. The *E_α_* of neat EP is 126.8 kJ/mol, while those of the composites first increase to 131.2 kJ/mol at 3% NiPS–NA and then decrease to 117.1 kJ/mol at excessive filler contents. The rise in activation energy is mainly attributed to well-dispersed NiPS–NA inorganic particles, which construct a stable reinforcing network inside the matrix and serve as a physical barrier to form tortuous diffusion paths, suppressing the volatilization of low-molecular-weight species and restricting the out-diffusion of pyrolysis products during thermal decomposition [47]. Nevertheless, the subsequent drop in *E_α_* originates primarily from the catalytic degradation effect of transition-metal species released from NiPS–NA, which accelerates chain scission of resin backbones and reduces the energy barrier for thermal decomposition at high temperatures [45]. More importantly, based on the variation in thermal decomposition parameters and previous literature regarding transition-metal-catalyzed epoxy pyrolysis [45], it is plausibly inferred that Ni^2+^-containing species may drive dehydrogenation and aromatization of EP pyrolysis fragments. Such a process could favor the generation of a compact, thermally stable char layer, which might partly account for the elevated T_max_ values of the composites. It should be noted that this catalytic pathway remains a qualitative speculation, and further characterization of pyrolysis residues will be required for direct experimental validation. The char yield of the composites shows a monotonic increase with increasing NiPS–NA loading. Neat EP delivers a char yield of only 16.1% at 700 °C, while the char yields of the composites rise steadily to 19.5–25.5% as the NiPS–NA content increases from 1% to 7%. This notable enhancement in char yield can be rationalized by two complementary factors. On the one hand, thermally stable inorganic NiPS–NA particles can persist as an inert reinforcing skeleton inside char residues. On the other hand, drawing from prior literature [45], Ni^2+^ transition-metal species are hypothesized to function as potential char-promoting sites, which may facilitate the transformation of organic pyrolysis products toward dense char rather than volatile small-molecule fragments, although such proposed char-catalyzing effect still awaits direct experimental confirmation via residue characterization. Consequently, the overall thermal stabilities of EP/NiPS–NA composites are significantly improved.

### 3.5. Tribological Properties of EP/NiPS–NA Composites

**Figure 6:**
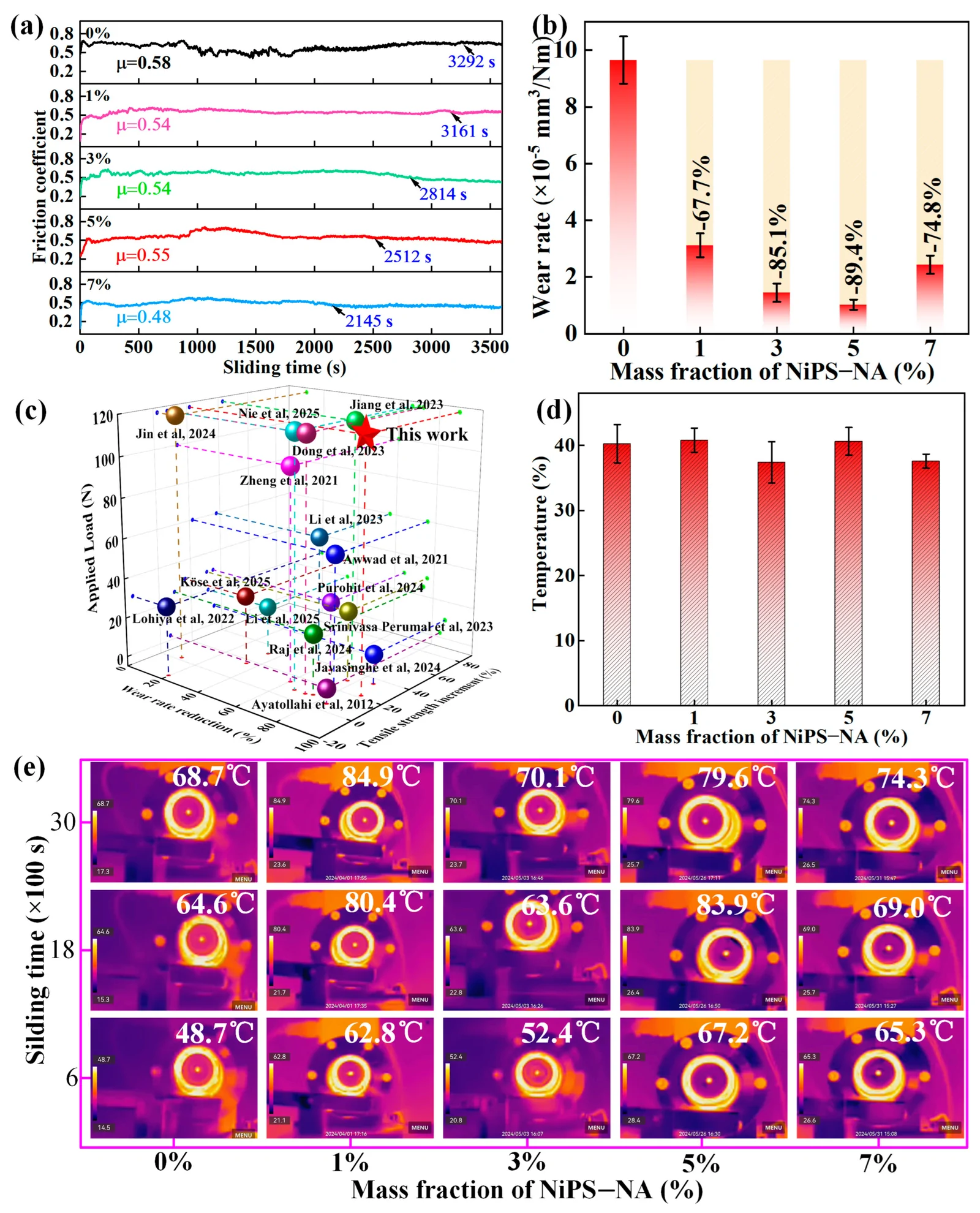
Dry-sliding properties of EP/NiPS–NA composites: (**a**) sliding curve, (**b**) wear rates of EP/NiPS–NA composites. (**c**) Comparison of anti-wear and mechanical properties [16,38,43,53,54,55,56,57,58,59,60,61,62,63,64], (**d**) average surface temperature of the contact surface and (**e**) infrared thermograms at varied sliding time.

Although materials with high tensile strength and surface hardness do not inherently guarantee outstanding anti-wear capacity, their intrinsic mechanical performance provides an important precondition for optimizing tribological behavior, and the actual friction and wear resistance cannot be simply deduced from static mechanical test results alone [48]. To systematically reveal the influence of NiPS–NA loading on the tribological performance of EP composites under starved lubrication conditions, a series of friction tests were carried out, with the corresponding results summarized in Figure 6. From the profiles of instantaneous friction coefficient versus sliding time in Figure 6a, all profiles feature an initial rapid increase in the instantaneous friction coefficient, followed by a relatively stable trend. Generally, the entire friction process can be divided into a running-in phase and a steady-state phase. In the running-in phase, actual contact occurs at tiny protrusions and pits on the uneven friction pair surface. The rapidly expanded real contact area and fractured transfer film usually cause drastic fluctuations in the instantaneous friction coefficient. The steady-state phase is dominated by the continuous formation of a stable solid transfer film between the material and friction pair [49]. It is clear that pure EP possesses the longest running-in phase with a duration of 3292 s and exhibits the highest average friction coefficient (μ) of 0.58. However, the running-in durations of the composites are progressively shortened to 2031~3161 s with increasing NiPS–NA content, and their average friction coefficients decrease to 0.48~0.54 depending on filler loading. This phenomenon indicates that incorporated NiPS–NA acts as a solid lubricant and improves the friction properties of EP composites to a certain extent. In particular, the 7% composite achieves the lowest μ value of 0.48, which is approximately 17.24% lower than that of pure EP. This finding is consistent with our previous work [16] but differs from other reported results, in which introduced phyllosilicate fillers increased the friction coefficient of EP composites [43]. This discrepancy is mainly attributed to the fact that friction-induced resin debris gradually accumulates in the contact zone and adheres to the surfaces of both rubbing pairs to form a transfer film, which transforms the contact mode from direct heterogeneous contact between the polymer and steel counterpart to homogeneous contact between transfer films [50]. More importantly, partial NiPS–NA detaches from the composite surface during friction and releases abundant inorganic nanosheets that participate in forming a denser and more continuous transfer film, thereby effectively alleviating direct contact between friction pairs [51]. Namely, NiPS–NA can efficiently facilitate transfer film formation, and this promotional effect becomes more pronounced at higher filler concentrations.

However, the wear rate, as depicted in Figure 6b, presents a different trend relative to the average friction coefficient. Pure EP possesses the maximum wear rate of 9.65 × 10^−5^ mm^3^/N·m, proving it has the poorest anti-wear performance among all the investigated samples. However, the incorporation of varied mass fractions of NiPS–NA suppresses the wear rate significantly, suggesting that the wear resistance of these composites can be remarkably improved. In fact, the wear rate of EP composites exhibits a typical trend of first decreasing followed by increasing with the continuous increase in NiPS–NA content. With the addition of 1% NiPS–NA, the wear rate of the composites declines remarkably to 3.12 × 10^−5^ mm^3^/N·m. Further increasing the NiPS–NA content leads to progressive reductions in the wear rate. When 5% NiPS–NA is added, a minimum wear rate of 1.02 × 10^−5^ mm^3^/N·m is achieved, corresponding to a maximum reduction of 89.4% compared with that of pure resin. This is attributed to the uniformly dispersed NiPS–NA that forms robust filler–matrix interfacial bonding with resin chains, which effectively hinders crack propagation and inhibits large-scale peeling of the resin matrix under cyclic friction loads [50]. Such a remarkable reduction in wear rate enabled by NiPS–NA demonstrates a superior reinforcing effect compared with previously reported results, in which pristine NiPS or MOF-derived bimetallic phyllosilicates exhibited limited wear rate suppression [16,52]. This discrepancy is closely related to the unique structural characteristics of NiPS–NA. Its loosely packed structure tends to disintegrate into abundant phyllosilicate nanosheets under compression–shear friction, which scatter over the friction contact surface and promote the formation of a robust, lubricious transfer film. This film isolates direct rigid contact between the composite specimen and steel counterpart, thereby alleviating abrasive mass loss. Nevertheless, at elevated NiPS–NA loadings, localized filler agglomerates detach from the matrix under prolonged shear stress and act as rigid abrasive debris, accelerating material erosion and causing a slight increase in wear rate. Even so, the final wear rate remains 74.8% lower than that of unfilled neat EP, which firmly validates the excellent anti-abrasion reinforcement efficiency of NiPS–NA in EP composites under the present dry-sliding conditions. Notably, the optimal filler loading differs for friction coefficient and wear rate, i.e., the 7% composite exhibits the lowest friction coefficient, while the 5% sample delivers the minimum wear rate. This discrepancy arises from competing effects of matrix reinforcement, transfer-film formation, local micro-agglomeration and third-body abrasion. At 5%, well-dispersed NiPS–NA effectively reinforces the EP matrix to suppress large-scale matrix peeling under cyclic shear. Sufficient released nanosheets construct a dense continuous transfer-film, contributing to the best anti-wear performance. At 7%, greater quantities of exposed nanosheet fragments enhance interfacial lubricity and lower friction. Nevertheless, local micro-agglomerates detach upon repeated shear to form rigid third-body abrasive debris. Even with a functional lubricious transfer-film, these particles accelerate material loss and increase the wear rate. In summary, friction coefficient is governed mainly by transfer-film lubricity, which favors higher filler content, whereas wear-resistance represents a balance between matrix reinforcement and agglomeration-induced third-body abrasion.

To benchmark the mechanical and tribological performance of the 5% NiPS–NA reinforced EP composite against State-of-the-Art systems, we collated published data on tensile-strength enhancement and wear-rate reduction for cross-system comparison, with the full results presented in Figure 6c and Table S3. The compiled dataset covers both recently reported filler-reinforced EP composites [53,54,55,56,57,58,59,60,61,62,63] and experimental findings from our prior works [16,38,43,64]. Despite the improved wear resistance offered by most existing EP composites, two common limitations can be generally identified. On one hand, satisfactory anti-wear performance usually depends on a relatively high filler loading [57,58,61,63]; on the other hand, many reported low wear rates are measured under substantially milder applied loads that deviate from practical heavy-duty service scenarios [53,57,58,61,62,63]. Notably, excessive filler incorporation tends to trigger internal mechanical defects within the resin matrix, which consequently gives rise to a remarkable decline in the overall mechanical strength [57]. As illustrated in Figure 6c, the EP/NiPS–NA composites fabricated in this work deliver a significantly higher degree of wear rate reduction than most comparable systems documented in available literature. This result clearly verifies the distinctive competitive advantage of the MOF-derived NiPS–NA in improving the wear resistance, underscoring its promising application potential and research value in the domain of polymer tribology.

Frictional heat accumulation is unavoidable during dry sliding and can soften the resin matrix, impair interfacial adhesion and accelerate material wear. Real-time temperature profiles varying with sliding time for all samples are presented in Figure S1. All specimens undergo a rapid temperature increase during the initial running-in phase, followed by slow temperature growth until quasi-stable thermal equilibrium is achieved after long-duration sliding. Random temperature fluctuations and sharp peaks along each curve correspond to transient flash temperatures generated by intermittent adhesion and micro-abrasion at friction interfaces [50]. As shown in Figure 6d, the average surface temperature of neat EP reaches a relatively high value of 40.6 °C after continuous sliding, indicating poor heat dissipation capacity. With the incorporation of NiPS–NA, the average surface temperatures of EP composites vary within a moderate range depending on filler content. This observation indicates that the introduction of NiPS–NA cannot induce substantial changes in the total frictional heat accumulated at the contact interface, failing to establish a straightforward correlation with the variation in wear rate. This result further demonstrates that the anti-wear performance of the composites is not merely governed by the average contact temperature; instead, it is jointly determined by multiple coupled factors, including interfacial contact stability, the continuity of the formed transfer film, matrix load-bearing capacity, and localized flash temperature accumulation within micro-contact regions [65]. Nevertheless, owing to the markedly higher thermal conductivity and heat capacity of the steel counterpart, the steel surface achieves a relatively elevated temperature during sliding, as clearly visualized by time-series infrared thermographs captured at different sliding durations in Figure 6e. These thermal snapshots further reveal that distinct localized hot spots persist within the contact zone throughout the sliding process, and the interfacial thermal distribution exhibits a pronounced dependence on both sliding duration and NiPS–NA filler loading.

### 3.6. Analysis of Wear Mechanism of EP/NiPS–NA Composites

**Figure 7:**
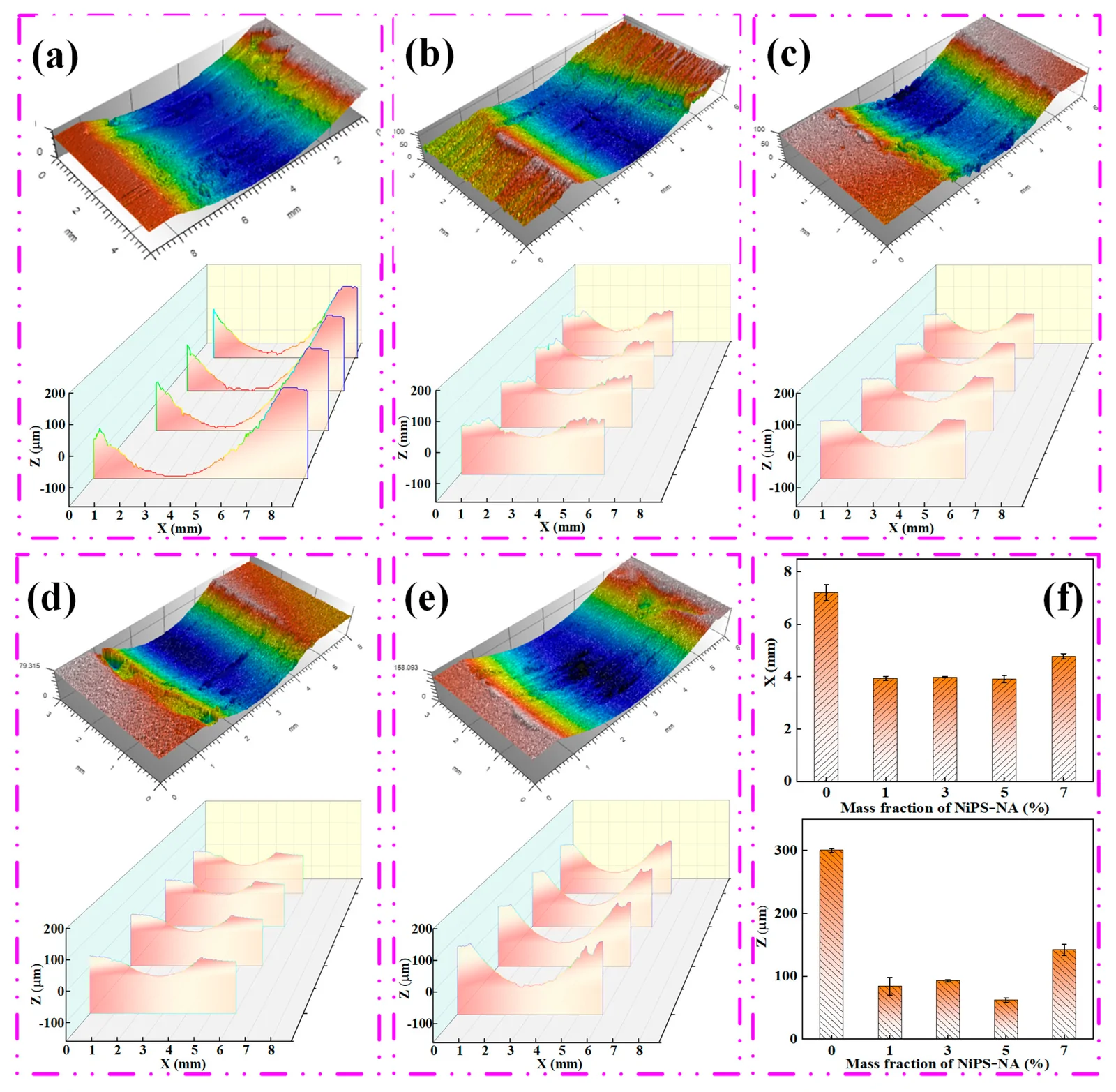
Three-dimensional morphologies and cross-section profiles of wear tracks for EP/NiPS–NA composites: (**a**) 0%, (**b**) 1%, (**c**) 3%, (**d**) 5% and (**e**) 7%; (**f**) variations in depth (X) and width (Z) versus NiPS–NA content.

To quantitatively clarify the evolution of wear-track morphology and systematically reveal the surface-damage mechanism of EP/NiPS–NA composites under dry sliding, three-dimensional laser scanning and SEM characterization were performed on the worn surfaces of all samples. The full 3D topographies and cross-sectional depth profiles of wear scars for neat EP and composites with different NiPS–NA loadings are displayed in Figure 7a–e, while the statistical variations in wear-track width (X) and depth (Z) are summarized in Figure 7f. All samples develop continuous arc-shaped wear tracks consistent with the contact profile of the steel counterpart, yet obvious differences in track depth and width are observed across different filler contents, which directly reflect the varying degrees of material abrasive loss under cyclic shear loading. Neat EP exhibits a maximum wear-track depth of 301.8 μm and the largest track width of 7.32 mm. In contrast, the incorporation of NiPS–NA markedly reduces the depth (Z) and width (X) of wear scars; the minimum values of 82.6 μm and 3.05 mm are obtained at 5% NiPS–NA loading, corresponding to reductions of 72.6% and 58.3%, respectively, compared with the unfilled sample. Although further addition of 7% NiPS–NA leads to a slight increase in wear-track depth and width, these values remain far lower than those of neat EP. Undoubtedly, such drastic reduction in the geometric dimensions of wear tracks provides intuitive morphological evidence for the prominent improvement in the wear resistance of EP composites, which matches well with the wear-rate variation trend discussed above.

**Figure 8:**
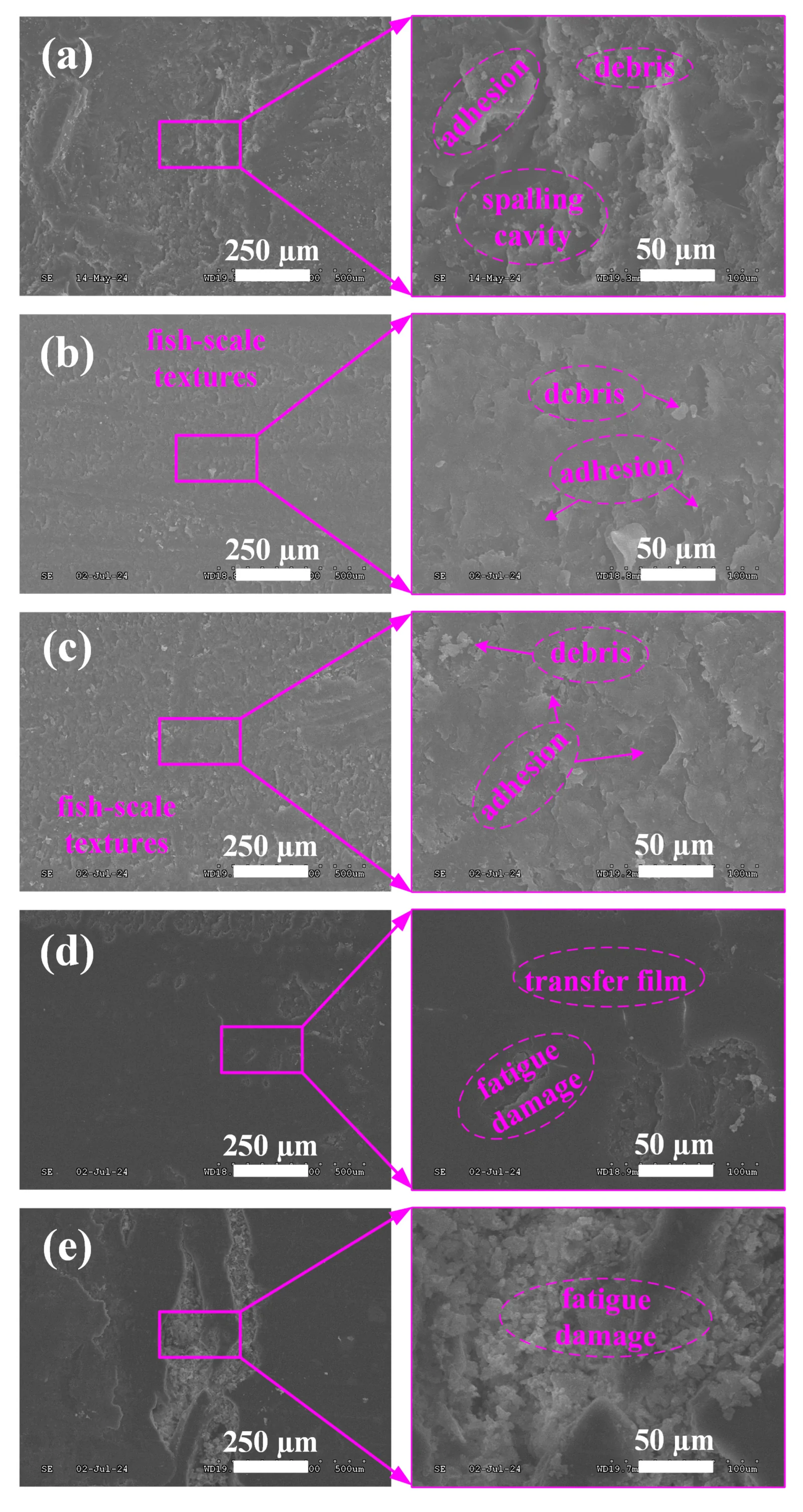
SEM images of worn surface for EP/NiPS–NA composites: (**a**) 0%, (**b**) 1%, (**c**) 3%, (**d**) 5% and (**e**) 7%.

To further unravel the dominant wear modes and underlying damage evolution, low- and high-magnification SEM micrographs capturing the worn surface features of all specimens are displayed in Figure 8. As shown in Figure 8a, the worn surface of neat EP is covered with abundant flaky resin fragments, deep spalling cavities and prominent adhesive wear marks. This severely irregular surface topography directly reveals the inferior abrasion resistance of unfilled EP, and adhesive wear coupled with fatigue damage serves as the primary failure mechanism under dry sliding [66]. Without NiPS–NA nanofillers acting as load-bearing skeletons and crack-deflection barriers, the highly crosslinked brittle EP matrix exhibits weak internal cohesion and limited resistance to cyclic frictional shear stress. Intensive plastic deformation readily occurs at the contact interface during reciprocating sliding, which hinders the stable formation of continuous protective transfer films between friction pairs. Thus, persistent matrix peeling and massive material loss occur throughout the friction process due to the absence of an effective lubricating transfer film that isolates rigid metal–resin contact. In sharp contrast, the incorporation of NiPS–NA induces a dramatic transformation in worn surface morphology. For specimens filled with 1% and 3% NiPS–NA (Figure 8b,c), the amounts of adhered resin debris and spalling pits decrease evidently, with only sparse shallow scratches distributed along the sliding direction. The worn surfaces become much smoother and display faint fish-scale textures, and the original coarse surface features are largely eliminated. Corresponding high-magnification images further confirm that spalling pits and craters are rare, and the degree of adhesive fracture is greatly alleviated. These morphological observations indicate that the rigid metal counterpart inevitably causes indentation and plowing damage on composite surfaces under repeated friction, which tends to compromise the structural integrity of the EP matrix. Nevertheless, appropriately dispersed NiPS–NA effectively hinders microcrack propagation under shear loading, significantly curbing resin shedding at the contact zone and reducing overall abrasion loss [44]. At the optimal loading of 5% NiPS–NA (Figure 8d), the sample presents a dense and continuous worn surface with only scattered minor abrasive traces. No large-scale resin shedding or severe adhesive failure is detected, and its contact surface is the smoothest among all test groups, indicating negligible material removal during dry sliding. Such exceptional wear resistance mainly stems from the uniformly distributed NiPS–NA within the EP matrix, which constructs rigid load-bearing networks to resist recurrent frictional shear and maintain the structural integrity of contact interfaces. More critically, detached NiPS–NA nanosheets continuously accumulate within the friction zone and assemble into a dense, intact protective transfer film to strongly restrain adhesive failure and nearly suppress fatigue-triggered matrix delamination [67], which ultimately enables the specimen to achieve the lowest wear rate among all tested samples. As the NiPS–NA content increases to 7% (Figure 8e), the overall worn topography remains relatively flat and uniform. However, fatigue damage traces become aggravated because excessive NiPS–NA introduces excessive brittleness, rendering the surface resin susceptible to frictional destruction. Neither obvious abrasive fragments nor large-scale matrix delamination is observed on the contact surface, demonstrating decent anti-abrasion performance and stable structural integrity of the friction interface.

**Figure 9:**
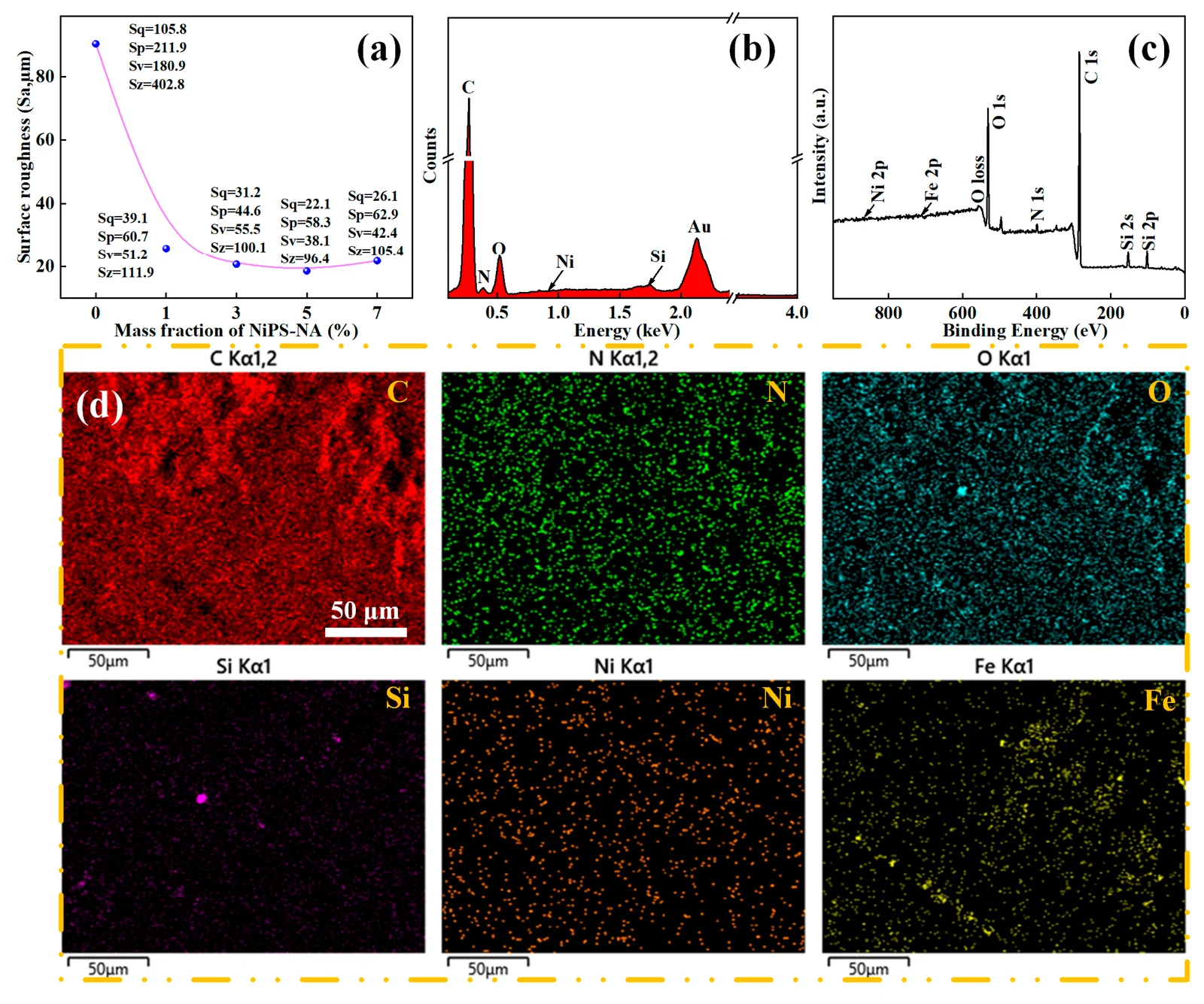
(**a**) Surface roughness versus the NiPS–NA content; (**b**) EDS pattern, (**c**) XPS survey scan and (**d**) elemental mappings of EP composites containing 5% NiPS–NA.

Distinct topographical differences among the wear tracks of all test specimens are further quantified via average surface roughness Sa, along with root-mean-square height Sq, maximum peak height Sp, maximum valley depth Sv and total height Sz. The statistical data for these roughness parameters are summarized in Figure 9a. Unfilled neat EP exhibits the maximum Sa value of 90.3 µm, which corresponds to its severely irregular, coarse worn surface formed after long-duration dry sliding. The incorporation of NiPS–NA drastically lowers the surface roughness, demonstrating a prominent improvement in the surface flatness of EP composites. When the NiPS–NA loading rises to 1% and 3%, the Sa values of the composites decrease sequentially to 25.9 µm and 20.7 µm, respectively. The specimen filled with 5% NiPS–NA exhibits the minimum Sa value of 18.6 µm, representing a maximum reduction of 79.4% compared with neat EP. This quantitative roughness result serves as additional direct evidence confirming that the 5% filler formulation yields optimal wear resistance. Although further elevating the NiPS–NA dosage to 7% triggers a mild rebound in Sa to 21.8 µm, this value remains markedly lower than that of pure EP. Collectively, the above roughness data solidly verify the morphological features of worn surfaces observed in SEM images and are in good accordance with the changing trend of wear rates discussed in the preceding section. The EDS spectrum presented in Figure 9b reveals the constituent elements involved in forming the solid transfer film. The characteristic C, N and O signals appearing at 0.27 keV, 0.39 keV and 0.53 keV, respectively, are primarily derived from the resin matrix, while weak Ni and Si peaks emerging at 0.87 keV and 1.74 keV originate from the incorporated NiPS–NA filler. An intense Au signal located at 2.12 keV arises from the gold conductive coating sprayed onto the sample surface, yet no characteristic iron (Fe) peak can be identified in the EDS spectrum owing to its low content and the instrument-detection limits. The full-range XPS survey spectrum in Figure 9c corroborates the above EDS results. Distinct strong peaks at 285 eV and 533 eV are assigned to C 1s and O 1s, while a weaker N 1s signal appears at 399 eV. The Si 2p and Si 2s characteristic bands are detected at 103 eV and 154 eV, respectively. In addition, Ni 2p and Fe 2p only exhibit faint signals at 853 eV and 711 eV, further verifying the extremely low abundance of these two elements within the transfer film. By contrast, the elemental distribution mappings in Figure 9d distinctly demonstrate the uniform dispersion of all target elements across the entire worn surface. Clear signals of Si, Ni and Fe can be captured within the abrasive contact zone on both the top surface and subsurface layers. These mapping results provide robust experimental evidence to confirm the aforementioned hypothesis that well-dispersed NiPS–NA fillers and captured Fe-containing debris from the steel counterpart jointly participate in constructing a dense, continuous solid transfer film, which correspondingly enhances the anti-abrasion capacity of EP composites.

**Scheme 2:**
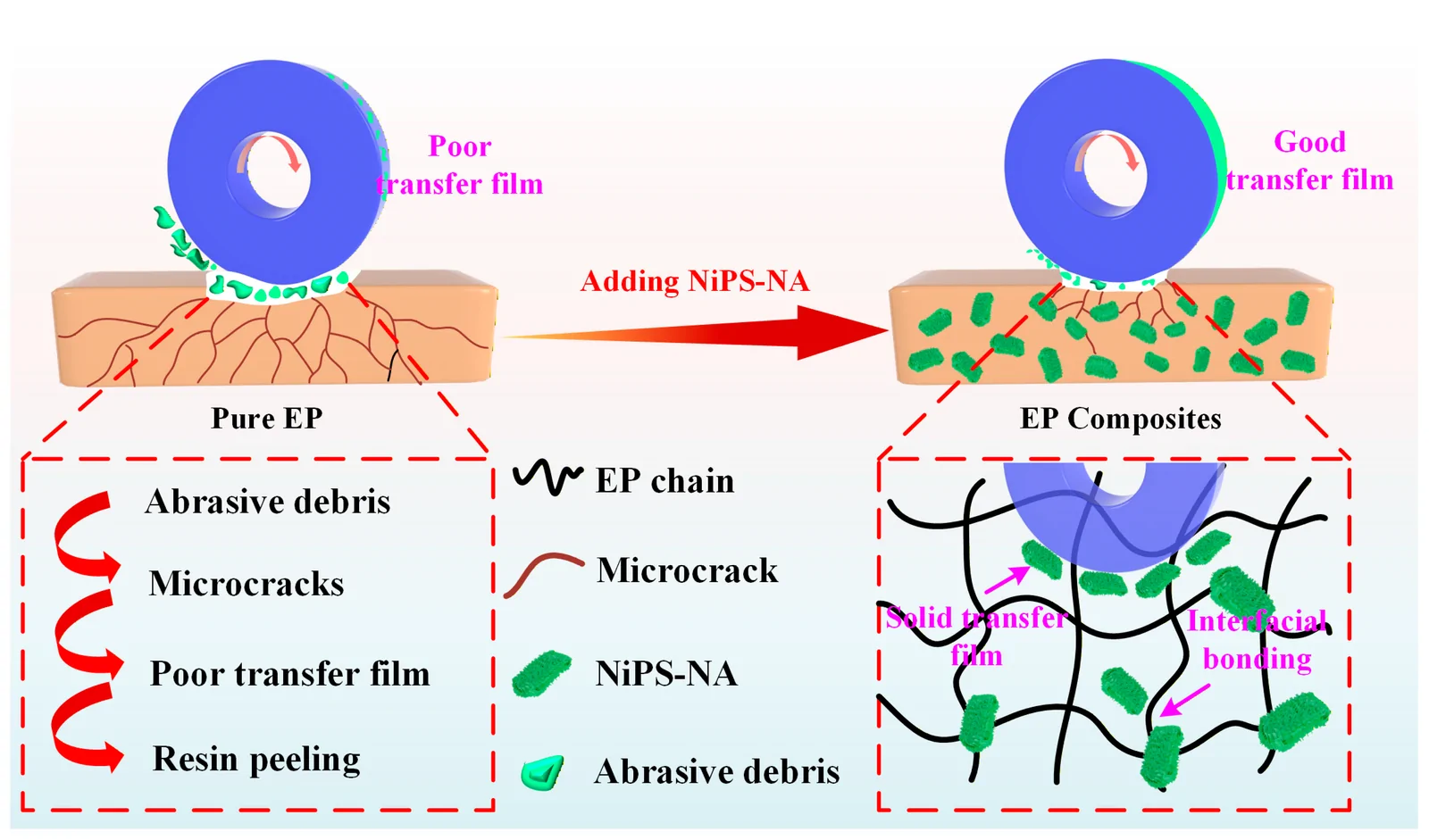
Schematic diagram revealing the wear mechanism of EP/NiPS–NA composites.

Based on the above analysis, the prominent amelioration in wear resistance derived from the appropriate incorporation of NiPS–NA is fully validated, and the corresponding wear mechanism of EP/NiPS–NA composites is illustrated in Scheme 2. Dry sliding is widely acknowledged as a time-dependent, multi-coupled complex process governed by mutual contact interactions between soft polymer specimens and rigid steel counterfaces. Under fixed sliding test conditions, the inherent microstructure and mechanical properties become the core factors controlling the tribological performance of materials. Neat EP exhibits low tensile strength and poor fracture toughness, rendering it susceptible to indentation, plowing and surface damage under cyclic compressive-shear loads. Persistent adhesive transfer of resin fragments to the counterface enlarges the actual contact area, while cyclic contact stress concentrates beneath the wear track to initiate and propagate subsurface microcracks within the matrix. When these internal cracks break through the top surface, unfilled EP undergoes combined mechanical–thermal fatigue wear under prolonged reciprocating sliding. Extensive surface cracking and abundant debris formation follow, ultimately causing severe material removal via continuous matrix spalling [68]. However, the appropriately incorporated NiPS–NA contributes significantly to the remarkable improvement in tribological performance from the following aspects. First, uniformly distributed NiPS–NA simultaneously improves the tensile strength and ductility of the EP matrix, suppressing severe plastic deformation under frictional shear, reducing the real contact area of friction pairs and raising the initiation threshold of fatigue cracks [69]. Moreover, NiPS–NA features loosely stacked nanosheets with a porous framework inherited from the Ni-MOF precursor and disperses uniformly in the EP matrix. Thus, abundant surface-active sites on NiPS–NA enable intensive physical entanglement with resin molecular chains to generate numerous strongly bonded filler–matrix interfaces. These interfaces act as effective crack deflectors, retarding crack extension, alleviating contact stress concentration and suppressing the development of interpenetrating crack networks in the EP substrate [70]. Finally, as the surface resin layer is gradually abraded, embedded NiPS–NA exposed on wear scars disintegrates into numerous nanosheets that accumulate throughout the contact zone. Combined with iron-bearing wear debris captured from the steel counterface, these nanosheets facilitate the generation of a dense, uninterrupted protective transfer film across the sliding interface, which provides effective shielding for the underlying EP matrix [71]. Accordingly, NiPS–NA-filled composites produce far less resin debris and exhibit superior resistance to cyclic frictional fatigue compared with neat EP.

## 4. Conclusions

In this work, a two-step hydrothermal strategy using Ni-MOF as a sacrificial precursor was developed to fabricate NiPS–NA, which was incorporated into EP as multifunctional nanofillers. Systematic characterizations were performed to clarify how NiPS–NA loading regulates the structure–property relationships of EP/NiPS–NA composites, and the underlying strengthening and anti-wear mechanisms were systematically elucidated.

Structural characterizations confirm the complete transformation of Ni-MOF into pure-phase, loosely stacked NiPS–NA nanoclusters, which disperse uniformly within the EP matrix without severe agglomeration. The mechanical properties exhibit distinct loading-dependent variations, achieving simultaneous improvements in tensile strength and ductility with increasing NiPS–NA content. The elongation at break of the composites follows a volcanic trend and peaks at 6.0% at 3% filler loading, corresponding to a 43.3% enhancement compared with neat EP. The tensile strength increases continuously, while the elastic modulus fluctuates slightly with elevated filler content. NiPS–NA exerts dual regulatory effects on the thermal decomposition behavior of EP/NiPS–NA composites. Appropriate NiPS–NA incorporation significantly reduces the thermal weight loss rate from 1.12%/K to 0.73%/K. Meanwhile, the char residue yield at 700 °C and thermal decomposition activation energy increase from 16.7% and 126.8 kJ/mol to 25.5% and 131.2 kJ/mol, respectively, effectively improving the overall thermal stability of EP composites. Under starved-lubrication dry-sliding conditions, NiPS–NA greatly enhances the tribological performance of EP composites. The wear rate decreases initially and then rebounds at higher filler loadings. The 5% NiPS–NA-filled composite achieves a minimum wear rate of 1.02 × 10^−5^ mm^3^/N·m, which is 89.4% lower than that of unfilled EP, while the average friction coefficient increases moderately and steadily. Comprehensive analysis of worn surfaces reveals that uniformly dispersed NiPS–NA delivers synergistic reinforcing effects and promotes the formation of dense and continuous transfer films, which protect the bulk substrate against abrasive deterioration.

This study establishes a facile structural design strategy for 2D layered nanofillers and provides a feasible pathway to fabricate high-performance tribological polymer composites, possessing great application potential for industrial wear-resistant components and surface protective coatings.

## Data Availability

The original contributions presented in this study are included in the article/Supplementary Materials. Further inquiries can be directed to the corresponding authors.

## Supplementary Materials

The following supporting information can be downloaded at: https://www.mdpi.com/article/10.3390/polym18182206/s1, Figure S1: Temperature profiles as a function of sliding time for EP composites with various mass fraction of NiPS-NA; Table S1: Sample formulae for the investigated samples; Table S2: Thermal parameters of EP/NiPS-NA composites; Table S3: Fitting results of samples based on Horowitz and Metzger equation; Table S4: Detailed comparison with previous reported works [72,73,74].

## Abbreviations

EP	Epoxy
MOF	Metal–organic frameworks
Ni-MOF	Nickel-based metal–organic frameworks
NiPS	nickel phyllosilicate
NiPS–NA	nickel phyllosilicate nanosheet assemblies
BN	Boron nitride
GO	graphene oxide
XRD	X-ray diffraction
FT-IR	Fourier-transform infrared spectroscopy
SEM	Scanning electron microscopy
TEM	Transmission electron microscopy
SAED	Selected-area electron diffraction
EDS	Energy-dispersive X-ray spectroscopy
HAADF	High-angle annular dark-field
XPS	X-ray photoelectron spectroscopy
TGA	Thermogravimetric analysis
DTG	Derivative thermogravimetric
