# Supplementary material for: Facilely Synthesis of MOF-Derived Nickel Phyllosilicate Nanosheet Assembly and Its Application in Robust, Toughened and Wear-Resistant Epoxy Composites
Source: Polymers (Basel). 2026 Sep 10;18(18):2206. doi: 10.3390/polym18182206 (PMC13611562; doi:10.3390/polym18182206)
Supplement: Supplementary file 1 [file polymers-18-02206-s001.zip › polymers-4544512-supplementary.pdf]

Supplementary Materials

Facilely Synthesis of MOF-Derived Nickel Phyllosilicate Nanosheet Assembly and Its Application in Robust, Toughened and Wear-Resistant Epoxy Composites

Dongliang Zhang [1,†], Weilong Chen [2,*,†], Hengzhi Zhou [3] and Jinian Yang [2,*]

1 School of Ocean Engineering, Guangzhou Maritime University, Guangzhou 510725, China
2 School of Materials Science and Technology, Anhui University of Science and Technology, Huainan 232001, China
3 School of Materials Science and Engineering, Nanjing Institute of Technology, Nanjing 211167, China
* Correspondence: chenweilong0535@163.com (W.C.) yangjinian@163.com (J.Y.)
† These authors contributed equally to this work.

2. Experimental

2.1 Raw materials and chemical agents

Nickel nitrate [$Ni(NO_3)_2·6H_2O$, 99%, AR], 1, 3, 5-benzenetricarboxylic acid (H_3BTC, 98%, AR), 4,4'-bipyridine ($C_{10}H_8N_2$, 99%, AR) and N,N-dimethylformamide (DMF, 99%, AR) were purchased from Shanghai Titan Scientific Co., Ltd (Shanghai, China). Sodium hydroxide (NaOH, 96%, AR), absolute ethanol (EtOH, 99.8%) and sodium silicate hydrate ($Na_2SiO_3·9H_2O$, ≥98%) were obtained from Aladdin (China). Tetrafunctional glycidylamine high-temperature resistant epoxy resin (EP, AG-80) was purchased from Hubei Zhengfeng New Materials Co., Ltd (Hubei, China). 1, 3-benzenedimethanamine (m-XDA, 99%, AR) was supplied by Shanghai Titan Scientific Co. Acetone (CH_3COCH_3, 99.5%, AR) was purchased from Western Chemical (Shanghai, China). Deionized water was self-contained in our laboratory. All chemicals were used as received without further purification.

2.2 Sample preparation

2.2 Sample preparation

The whole procedure of sample preparation briefly consists of three major steps, with the entire pathway illustrated in Scheme 1. ***(1) Synthesis of Ni-MOF***. 0.4362 g of $Ni(NO_3)_2·6H_2O$, 0.3165 g H_3BTC and 0.288 g 4,4'-bipyridine were dissolved in 60 mL of DMF, ultrasonicating until their completely dissolved. The obtained homogeneous solution was transferred into a 100 mL PTFE-lined autoclave, sealed and placed in an oven, reacting at 120 °C for 24 h. After cooling down naturally to ambient temperature, the product was collected by repeated centrifugation, washing with DMF and EtOH in sequence, and finally dried to a constant weight under vacuum at 80 °C to obtained green powder of Ni-

MOF. ***(2) Synthesis of NiPS-NA***. 0.4 g Ni-MOF and 0.57 g $Na_2SiO_3·9H_2O$ were added into 60 mL EtOH/H_2O solution (1:1 in volume) under strong stirring magnetically, and the 3 mL NaOH solution (1 mol/L) was further added to form a suspension. After sonication for 30 min, the mixture was carefully transferred into a PTFE-lined autoclave and hydrothermally reacted at 160 °C for 15 h. After severally repeated centrifugation and washing alternately with EtOH and deionized water, and finally dried to a constant weight under vacuum at 70 °C, the target light green NiPS-NA powders were achieved. ***(3) Fabrication of EP composites***. Pure EP and the composites were fabricated exactly following the detailed process described in detail in our previous report [72]. The concentration of NiPS-NA was ranged from 1% to 7% in mass fraction related to EP matrix.

Table S1 Sample formulae for the investigated samples

Sample	AG-80 (g)	m-XDA (g)	NiPS-NA (g)
0%	10	2.6	0
1%	10	2.6	0.1
3%	10	2.6	0.3
5%	10	2.6	0.5
7%	10	2.6	0.7

2.3 Performance testing

2.3.1 Structural characterization

The crystal structure was analyzed by an X-ray diffractometer (XRD-6000, Shimadzu), and the relevant information was collected by radiation using a CuK_{α} target at an acceleration voltage of 40 kV. The molecular structure and chemical bonding were characterized by Fourier Transform Infrared Spectroscopy (FT-IR, Nicolet380, Thermo Scientific) in the range of 400-4000 cm^{-1}. The scanning electron microscopy (SEM, S3000 N and Regulus 8100, HITACHI) and transmission electron microscopy (TEM, JEM 2100 PLUS, JEOL) were used to record the morphological structures. The elemental composition and distribution were detected through energy dispersive spectroscopy (EDS) and x-ray photoelectron spectroscopy (XPS, ESCALAB Xi^+, Thermo Scientific).

2.3.2 Performance testing

At room temperature, the dumbbell-shaped samples were subjected to tensile tests using a universal testing machine (WDW-50, KQL) under a cross-section rate of 2 mm/min, and each formula was duplicated at least 5 times. Surface hardness was conducted using a Shore D hardness tester (LXD-D, SANLIANG), with reported values based on at least 10 duplications. The tribological response was

evaluated using a plastic friction and wear testing machine (M200, ZHONGLU) without any lubrications performed following the previous report [52]. The real-time temperature of the sample during the friction process was recorded by a non-contact infrared thermometer (ABSD-01A, ABOSUD Company) and image was captured using a hand-held thermographic camera (HM-TPK20-3AQF/W). Thermogravimetric analysis (TGA, Q500, TA INSTRUMENTS) was used to analyze the thermal stabilities of investigated samples, with a heating rate of 10 °C/min. After tribological tests, the worn surfaces of investigated samples were measured using a non-contact three-dimensional optical profiler (PS50, NANOVEA), obtaining the results of topographic images and cross-sectional profile. Besides, another important indicator for characterizing the wear grooves, surface roughness (Sa), is also determined according to ISO 25178, which includes four parameters: maximum height (Sz), root-mean-square height (Sq), maximum crest height (Sp) and maximum degree of concavity (Sv).

Figure

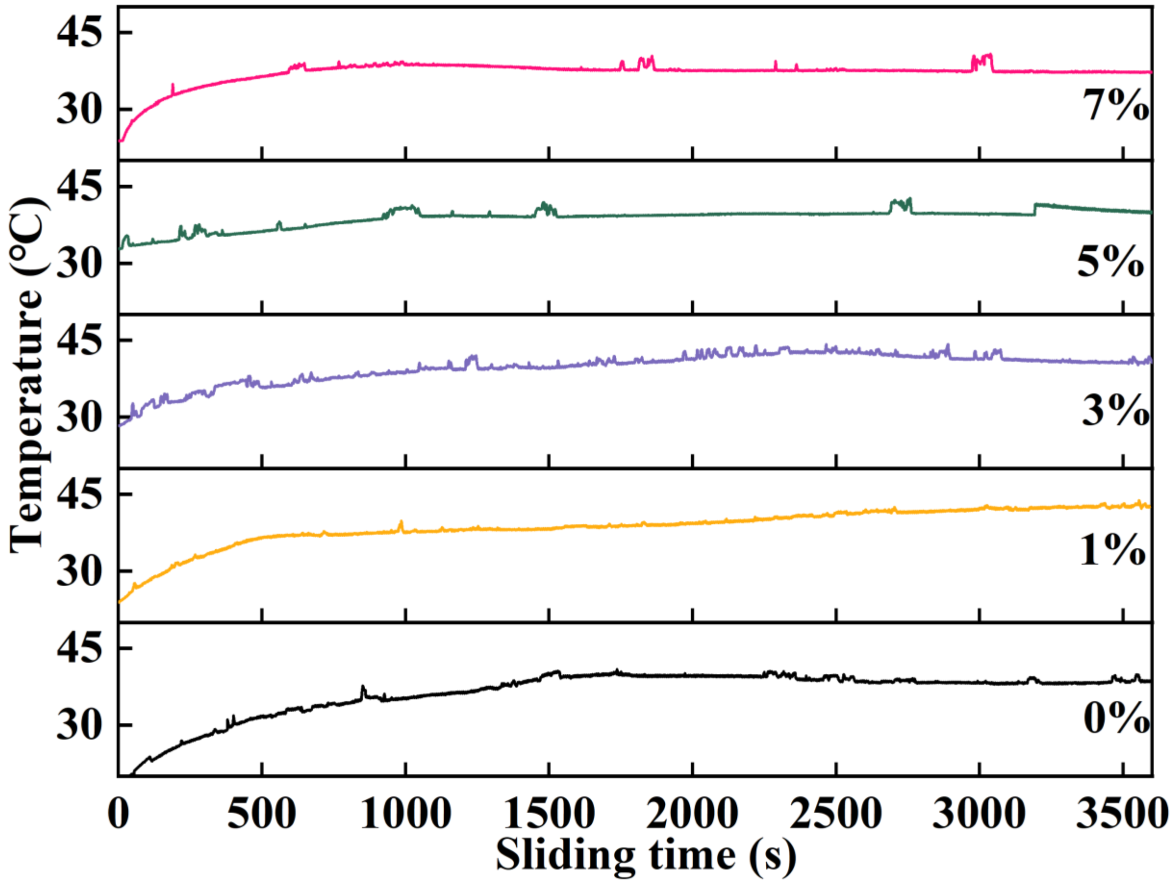

Figure S1 Temperature profiles as a function of sliding time for EP composites with various mass fraction of NiPS-NA

Table

Table S2 Thermal parameters of EP/NiPS-NA composites

Sample	$T_{5\%}$ (°C)	T_s (°C)	T_{max} (°C)	$(dW/dT)_{max}$ (K/%)	E_α (kJ/mol)	Char (%)
0%	322.7	173.5	379.6	1.12	126.8	16.7
1%	311.7	172.7	380.8	0.86	123.3	19.5
3%	321.5	178.6	410.7	0.83	131.2	19.7
5%	303.8	169.9	373.6	0.73	127.3	22.3
7%	299.3	170.6	384.8	0.89	117.1	25.5

Note: $T_{5\%}$ represents the temperature at which the weight loss fraction reaches 5%, commonly defined as the onset decomposition temperature. Similarly, T_{max} denotes the temperature corresponding to the maximum decomposition rate, with $(dW/dT)_{max}$ being the corresponding mass loss rate. The char content is the mass fraction of residual solid matter after thermal decomposition at 700 °C. E_α is the activation energy of thermal decomposition determined using a calculating procedure proposed by Horowitz and Metzger [73] and expressed in Equations (S2)~(S3), where $T_{30\%}$ means the temperature when the weight loss fractions is up to 30%, α represents the weight conversion fraction at a temperature of T, and R is the gas constant. T_s is the statistical heat resistance index of the composites in terms of thermal fluidity and thermal stability, which is employed to reflect the resistance to heat flow before it is thermally damaged and calculated according to Equation (S1) [74].

$$T_S = 0.49[T_{5\%} + 0.6(T_{30\%} - T_{5\%})] \quad (S1)$$

$$\ln[\ln(1-\alpha)^{-1}] = \varphi \frac{E_\alpha}{RT_{max}^2} \quad (S2)$$

$$\varphi = T - T_{max} \quad (S3)$$

Table S3 Fitting results of samples based on Horowitz and Metzger equation

Sample	Slop	Intercept	Fitted equation	Pearson's r
0%	0.03577	-0.8761	y=0.03577·x-0.8761	0.992
1%	0.03196	-0.8438	y=0.03196·x-0.8438	0.987
3%	0.02901	-0.9201	y=0.02901·x-0.9201	0.988
5%	0.02828	-1.0492	y=0.02828·x-1.0492	0.988
7%	0.03063	-1.0100	y=0.03063·x-1.0100	0.989

Table S4 Detailed comparison with previous reported works

Sample	Mass fraction of filler	Wear rate reduction	Tensile strength increment	Load/speed/duration	Ref.
EP/MoS_2	0.3%	86.3%	35.0 %	10 N/0.2 $m\cdot s^{-1}$/2100 s	[53]
EP/GNPS	4.5%	76.0 %	22.0 %	60 N/2 $m\cdot s^{-1}$/3000 s	[54]
EP/NC	1.5%	60.3 %	13.7 %	100 N/ 0.1 $m\cdot s^{-1}$/7200 s	[55]
EP/CPB	5.0%	75.0 %	9.5 %	25 N/0.52 $m\cdot s^{-1}$/4800 s	[56]
EP/Slag	40 %	19.6 %	-15.6 %	30 N/1 $m\cdot s^{-1}$/3000 s	[57]
EP/WAD	12%	65.0%	34.7%	30N/0.3 $m\cdot s^{-1}$/1000 s	[58]
EP/ND	0.1%	84 %	6.2 %	2.5 N/1 $m\cdot s^{-1}$/800 s	[59]
EP/MWFB	15%	34.6%	16.4%	30 N/0.4 $m\cdot s^{-1}$/2500 s	[60]
EP/$FeTiO_3$/SiO_2	5%	76.7 %	30.5 %	30 N/0.96 $m\cdot s^{-1}$/104s	[61]
EP/BF/MOF/PW	1%	56.8 %	37.6 %	60 N/0.42 $m\cdot s^{-1}$/1800 s	[62]
EP/C-CNTs/CF	55%	34.4 %	31.2 %	20 N/ 0.06 $m\cdot s^{-1}$/3600 s	[63]
EP/NiFePS	1%	10.2 %	2.3%	117.6 N/1.05 $m\cdot s^{-1}$/3600 s	[38]
EP/PANI	5%	70.0 %	43.0%	117.6 N/1.05 $m\cdot s^{-1}$/3600 s	[37]
EP/HN-NiPS	3%	72.1 %	8.2%	117.6 N/1.05 $m\cdot s^{-1}$/3600 s	[43]
EP/K-NiPS/DOPO	5%	66.7 %	8.2%	117.6 N/1.05 $m\cdot s^{-1}$/3600 s	[64]
**EP/NiPS-NA**	5%	89.4 %	21.8%	117.6 N/1.05 $m\cdot s^{-1}$/3600 s	**This work**

Note MoS_2: Molybdenum disulfide; GNPs: Graphene nanoplatelets; NC: Nanoclay; CPB: Cassava periderm biosilica; WAD: Wood apple dust; ND: Nanodiamond; MWFB: Modified waste fish bone; $FeTiO_3$: Ilmenite; SiO_2: Silicon dioxide; BF: Basalt; MOF: Mental organic framework; PW: Paraffin wax; C-CNTs: Carbon nanotube; CF: Carbon fiber; NiFePS: Nickel iron phyllosilicate; PANI: Polyaniline; HN-NiPS: Hierarchical nanosheets of nickel phyllosilicate; K-NiPS: MOF-derived nickel phyllosilicate; DOPO: 9,10-dihydro-9-oxa-10-phosphaphenanthrene-10-oxide.
